# Pathogenic nsSNPs that increase the risks of cancers among the Orang Asli and Malays

**DOI:** 10.1038/s41598-021-95618-y

**Published:** 2021-08-09

**Authors:** Nurul Ain Khoruddin, Mohd NurFakhruzzaman Noorizhab, Lay Kek Teh, Farida Zuraina Mohd Yusof, Mohd Zaki Salleh

**Affiliations:** 1 Integrative Pharmacogenomics Institute (iPROMISE), Universiti Teknologi MARA (UiTM), Selangor Branch, Puncak Alam Campus, 42300 Puncak Alam, Selangor, Malaysia; 2 Faculty of Applied Sciences, Universiti Teknologi MARA (UiTM), Shah Alam Campus, Selangor, Malaysia; 3 Faculty of Pharmacy, Universiti Teknologi MARA (UiTM), Selangor Branch, Puncak Alam Campus, 42300 Puncak Alam, Selangor, Malaysia

**Keywords:** Cancer, Computational biology and bioinformatics, Medical research, Oncology, Risk factors

## Abstract

Single-nucleotide polymorphisms (SNPs) are the most common genetic variations for various complex human diseases, including cancers. Genome-wide association studies (GWAS) have identified numerous SNPs that increase cancer risks, such as breast cancer, colorectal cancer, and leukemia. These SNPs were cataloged for scientific use. However, GWAS are often conducted on certain populations in which the Orang Asli and Malays were not included. Therefore, we have developed a bioinformatic pipeline to mine the whole-genome sequence databases of the Orang Asli and Malays to determine the presence of pathogenic SNPs that might increase the risks of cancers among them. Five different in silico tools, SIFT, PROVEAN, Poly-Phen-2, Condel, and PANTHER, were used to predict and assess the functional impacts of the SNPs. Out of the 80 cancer-related nsSNPs from the GWAS dataset, 52 nsSNPs were found among the Orang Asli and Malays. They were further analyzed using the bioinformatic pipeline to identify the pathogenic variants. Three nsSNPs; rs1126809 (TYR), rs10936600 (LRRC34), and rs757978 (FARP2), were found as the most damaging cancer pathogenic variants. These mutations alter the protein interface and change the allosteric sites of the respective proteins. As *TYR*, *LRRC34,* and *FARP2* genes play important roles in numerous cellular processes such as cell proliferation, differentiation, growth, and cell survival; therefore, any impairment on the protein function could be involved in the development of cancer. rs1126809, rs10936600, and rs757978 are the important pathogenic variants that increase the risks of cancers among the Orang Asli and Malays. The roles and impacts of these variants in cancers will require further investigations using in vitro cancer models.

## Introduction

Single nucleotide polymorphisms (SNPs) are the major type of genetic variation in humans (~ 90%). Thus far, around 500,000 SNPs have been reported on the coding regions of the human genome^1^. Among these, the nonsynonymous SNPs (nsSNPs) change the residues of amino acids of the protein sequences and may have damaging or neutral effects on the protein functions or structures^2,3^. Damaging nsSNPs may affect the function or structure of a protein by modifying the protein charge, geometry, hydrophobicity^4^, stability, dynamics, translation, and protein interactions^5,6^. These are probably the significant factors that contribute to the functional diversity of encoded proteins in the human population^7^. Therefore, many human diseases could be due to these damaging nsSNPs.

Previous studies have shown that nsSNPs cause numerous genetic disorders such as inflammatory and autoimmune disorders and cancers^8–10^. With the massive human genome sequence data now available and we are yet to know the functional effects of some of the SNPs, a more cost-effective approach is required to unravel the functions of the unknown SNPs effects. Many studies have used bioinformatics tools to predict the deleterious effects of nsSNPs on the functions of proteins that result in diseases before expensive in vitro or in vivo experiments are conducted. Two nsSNPs on the *ABCB1* gene had been associated with breast cancer, and these SNPs were predicted for their deleterious effects, which caused the change in protein conformation using comprehensive bioinformatics analysis^11^. A similar study using functional and structural bioinformatics tools had identified three damaging nsSNPs that alter the functions and structures of the *RNASEL* gene. These nsSNPs are most likely pathogenic and associated with the increase of prostate cancer susceptibility^12^. nsSNPs in the *KRAS* gene have been found to be associated with lung cancer due to their damaging effects on the structural features of the protein. The structure and function of the native proteins were found to be altered due to the nsSNPs using a pipeline comprised of several bioinformatics tools^13^. A recent study had identified the deleterious nsSNPs on the *hOGG1* gene that altered the secondary structure of the expressed protein and destabilized its local conformation, which increases the risks for lung cancer^14^. Furthermore, *in-silico* modeling has been widely used to assess the functional impacts of nsSNPs and their possible roles in cancers^15,16^. *in-silico* modeling has the advantage of being able to make rapid predictions for the mechanisms of actions of a wide range of compounds in a high-throughput mode. Another advantage is that prediction can be made based on the structure of a compound before it issynthesized^17^.

Databases of human variants have been developed with different scopes and contents used to predict diseases^18^ in achieving personalized medicine^19^. The genome-wide association study (GWAS) database (https://www.ebi.ac.uk/gwas/) is widely used to associate SNPs with diseases. Although there are other existing human variant databases such as ClinVar, COSMIC, SwissVar, and Humsavar, GWAS is the only database that gives a world of information or catalogs on disease mutations in different populations. This database also provides information on the statistically significant variants and the increase/decrease associated risks for each phenotype^20^.

The application of genomics, bioinformatics, and the availability of data generated from high-throughput technologies are the fundamental tools for implementing precision medicine not only for cancer diseases but also for other common and rare diseases^21,22^. Various tools have been used to predict the functional effects of nonsynonymous coding variants using basic sequence homology^23–25^; empirically derived rules^26^; structural and functional features^27–29^; a weighted average of the normalized scores^30^; decision trees^31,32^; support vector machines^33–36^; and Bayesian classifiers^27^. A comprehensive systematic evaluation study on the performances of these widely used prediction methods to identify the pathogenicity of the SNPs is required^37^. While new and more algorithms are being developed, the accuracy of prediction using a combination of the different algorithms should be validated. It is recommended that different computational methods are used to determine the impact of different SNPs during the screening step, and further validation should be incorporated in studying the impacts of nsSNPs on specific proteins^38^. In addition, complementary methods could be combined in a meta-server to yield more reliable predictions^39^. Several recent studies had reported on the use of a combination of various methods to uncover the potential impact of the nsSNPs in understanding the molecular mechanisms of various diseases, which includes cancers^40–44^. The combination of these tools allows more accurate prediction using the multiple conservation, structural, or combined methods (conservation and structural). Therefore, combined methods and meta-prediction methods (predictors that integrate multi-predictor results) are important for biomedical applications. This is because they can be applied to a much greater number of single nucleotide variants, considering that many human proteins do not currently have an experimentally defined structure or a close homolog to construct a model. Thus, combined and meta-prediction methods have a wide range of potential applications using the combinations of features yet to be explored^45^. As GWAS is usually conducted on a large population size using a high throughput detection method and is costly, some world populations were not studied. Therefore, their disease risks are not available. The Orang Asli are still practicing traditional healing methods, therefore the record on the incidence of cancers among the Orang Asli is lacking. This has posed challenges to the authorities to strategize health programs to ensure the sustainability of the Orang Asli. Due to the lack of phenotypic data on cancers, mining the genomes of the Orang Asli to predict their susceptibility for the different types of cancers would provide important data that allows the scientists to strategize research focus areas and for the authorities to provide relevant funding. In this study, we aimed to develop and validate a bioinformatics pipeline to detect and annotate the cancer-associated nsSNPs of a genome database and predict the structural and functional impacts of these nsSNPs that might increase the risks of cancers among the Orang Asli. Using the same pipeline, we also investigate the cancer risks of the Malays, which constitute the biggest population in Malaysia. The database of the Malay genomes was provided by Wong et al.^46^ and lacks information on the phenotypic traits, therefore it is interesting to predict the cancer susceptibility risks for this cohort using the established pipeline. The pipeline is developed using multiple bioinformatic tools in order to analyze the most deleterious and damaging nsSNPs associated with cancers. It includes the steps used for mining and annotating the genotypes and in silico* modeling* to predict the structural and functional impacts of the genetic variants with unknown functions. The new variants with potential impacts would be subsequently investigated in our laboratory using zebrafish models, and genotyping methods targeting the nsSNP would be developed for population study. In this study, three-dimensional (3D) protein models of the native and their variants (or mutant) were prepared. This is the first report which covers a comprehensive in silico analysis of three (3) nsSNPs, rs1126809, rs10936600, and rs757978 for TYR, LRRC34, and FARP2 proteins, respectively. This study is a part of our initiatives to enhance precision health in our country. The bioinformatics pipeline developed in this study will be used in the future to predict genomic variations associated with different diseases.

## Methods

### Whole genome sequences

The whole-genome sequences of ninety-eight (98) healthy and unrelated Orang Asli from six different sub-tribes were retrieved from the Whole-Genome-Sequence Database at Integrative Pharmacogenomics Institute (iPROMISE) in the form of a bam file. The Orang Asli were recruited from sub-tribes that are located in the (i) northern region of the Peninsular Malaysia [( Bateq, n = 22; Gua Musang, Kelantan), ( Lanoh, n = 16; Lenggong, Perak) and ( Kensiu, n = 19; Baling, Kedah); (ii) in the central region [(Che Wong, n = 18; Kuala Gandah, Pahang) and (Semai, n = 16; Kuala Lipis, Pahang); in the southern region [(Kanaq, n = 7; Kota Tinggi, Johor)]. The mean coverage of whole-genome sequences of Orang Asli across all the 98 samples was 37.39 × (minimum of 18.44 × to a maximum of 46.02x) and was checked using Qualimap version 2.2.1.

The genomic DNA (gDNA) of each of the 98 Orang Asli individuals was isolated from 300 µl of whole blood using the Wizard Genomic DNA Purification Kit (Promega, Wisconsin, USA). A microvolume spectrophotometer (NanoDrop 2000, Thermo Scientific) was used to evaluate DNA quantity. Whole-genome sequencing of the 98 Orang Asli were then performed using the Genome Analyzer System (GA IIx) with a target of > 30 × coverage. The whole-genome sequences of Orang Asli were then assembled by the in-house bioinformatics workflow. Quality on the raw sequence data was checked with FastQC (https://www.bioinformatics.babraham.ac.uk/projects/fastqc/) and trimmed with Trimmomatic version 2.5^96^ (https://software.broadinstitute.org/gatk/best-practices/) recommended by the Genome Analysis Toolkit (GATK) Best Practices. Briefly, the reads were aligned using BWA version 0.6.1-r104 to the reference human genome GRCh37/hg19^97^ and duplicates were labeled and extracted using Picard version 1.119 (http://broadinstitute.github.io/picard/).

Whole-genome sequences of ninety-six (96) healthy Singaporean Malays were obtained in the form of bam files from Singapore Sequencing Malay Project (http://www.stategen.ns.edu.sg/~SSMP)^46^. Malays are Austronesians-speaking ethnic group who mainly live in Malaysia, Indonesia, and Singapore in the Southeast Asian region^46^. The mean coverage of the whole-genome sequences of Singapore Malays across all the 96 samples was 47.6x. The depth of coverage for each sample ranged from 35.5 × to 81.9x. All the genomic DNA of 96 Malays individuals was collected from the Singapore BioBank. Picogreen was used to measure fluorescence intensity, and the SpectraMax Gemini EM microplate reader was used to confirm that the DNA content was greater than 50 ng/l using spectrophotometric settings at 480/520 nm (Ex/Em). Subsequently, DNA samples were sent to the Defense Medical and Environmental Research Institute for preparation. Whole-genome sequencing of 96 Malays were then performed using the Illumina HiSeq 2000 with a target of > 30 × coverage.

Variant calling pipeline was performed using HaplotypeCaller and BaseRecalibrator (GATK v2.5)^98^ for each sequence data (bam file format) of the Orang Asli and Malays. The HaplotypeCaller was used to detect variants and BaseRecalibrator was used for base quality score recalibration (BQSR). Vcf files for each sample were generated for quality-filtering. Variant filtering was performed using SelectVariants (GATK v2.5)^98^, to extract SNPs and exclude variants with a read depth of less than 5 or a quality Phred score of less than 30.

The study protocol was approved by Universiti Teknologi MARA Research Ethics Committee [600-RMI (51/6/01) & 600-RMI (5/1/6)] and the Department of Orang Asli Development (Jabatan Kemajuan Orang Asli Malaysia, JAKOA) Research Ethics Committee [JAKOA.PP.30.052 Jld 5(62)].

### Bioinformatics workflow

**Figure 1:**
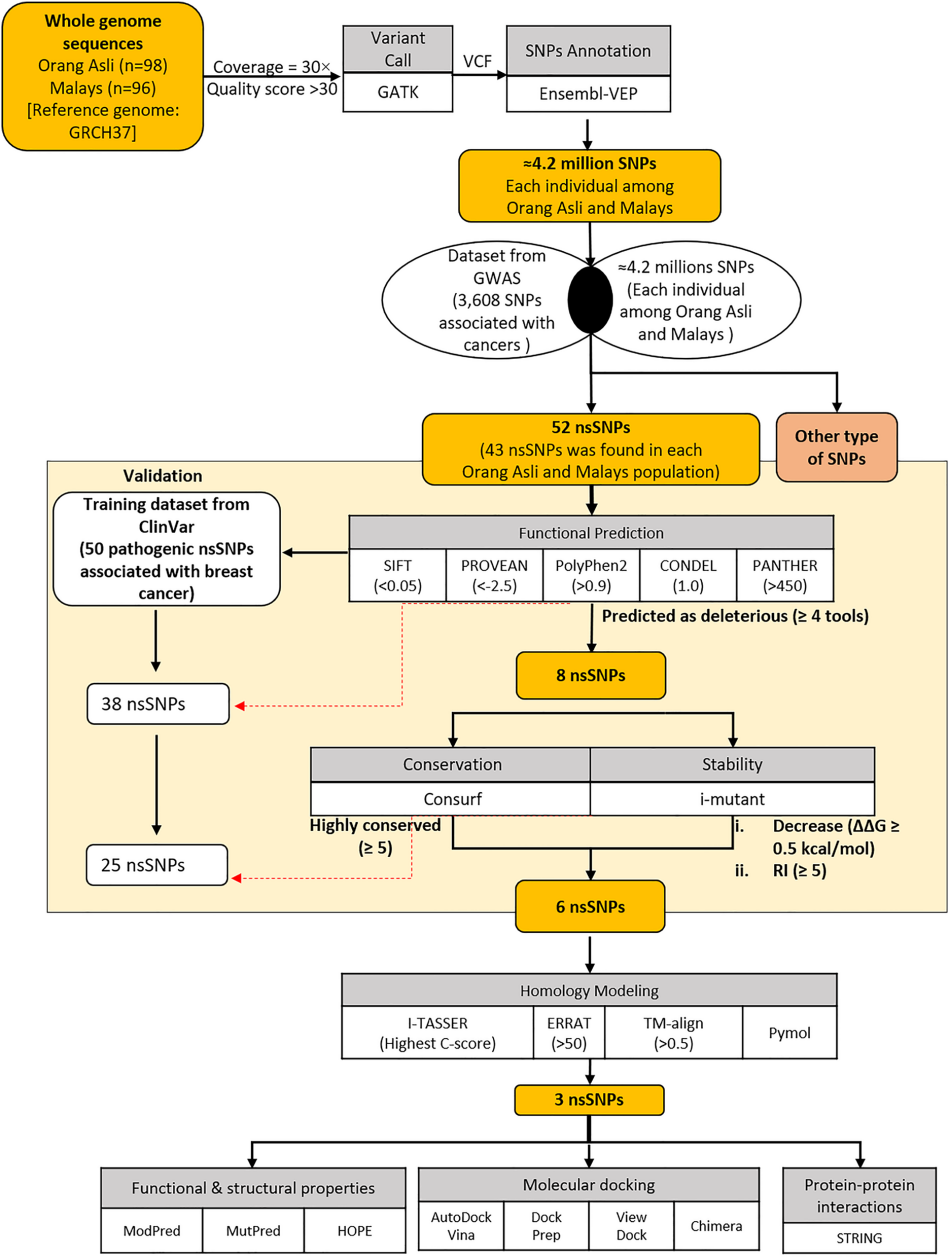
A workflow diagram for predicting high-risk cancer-related nsSNPs. The training dataset used was ClinVar to validate the capability of the pipeline to identify pathogenic variants based on the prediction of functional effect, conservation, and stability of cancer-related variants reported in Clinvar. The red dotted line represented the results for the training dataset.

High-risk nsSNPs associated with cancer were classified using the GWAS-Catalog as the source of the dataset, and various bioinformatics tools were employed in the workflow (Fig. 1).

### Nonsynonymous SNPs datasets for validation

The sensitivity, specificity, and accuracy of the functional effect prediction were determined using a combination of five different algorithms (SIFT, PolyPhen-2, Condel, PROVEAN, and PANTHER), with and without conservation (Consurf) and protein stability (I-Mutant). The standard dataset used comprised of nsSNPs associated with breast cancer from ClinVar. The ClinVar dataset includes a total of 100 clinically tested nsSNPs in which 50 nsSNPs were reported as pathogenic while the other 50 nsSNPs were reported as benign (Table S1). The 100 nsSNPs training dataset were randomly chosen out of 1020 clinically tested nsSNPs associated with breast cancer reported in the ClinVar as it is one of the most commonly studied cancer dataset. Although the dataset is primarily associated with breast cancer, the main purpose of using the training dataset is to test the ability of the pipeline to detect all the deleterious nsSNPs. Additionally, the sample size chosen also is sufficient as concluded by Thusberg et al., that the analysis result of using a small dataset (100SNPs) is comparable to a larger size (1000 SNPs) for a training dataset^37^. Datasets of different types of cancer and a larger sample size may also be used to achieve the same objective.

Analytical parameters of studied tools were calculated using Eqs. (1), (2), and (3) according to Fletcher^99^ and Glantz^100^.

Sensitivity (Se) is a proportion of the true-positive results (correct identification of pathogenic variants), according to Eq. (1).

1 $$ Se = \frac{TP}{{TP + FN}} \times 100\% $$

where TP denotes true-positive cases, and FN denotes false-negative cases.

Specificity (Sp) is a proportion of the true negative results (correct identification of benign variants), according to Eq. (2).

2 $$ Sp = \frac{TN}{{TN + FP}} \times 100\% $$

where TN denotes true negative cases, and FP denotes false-positive cases.

Accuracy (Ac) is the ratio of complete, correct predictions to the total number of predictions, according to the following Eq. (3).

3 $$ Ac = \frac{TP + TN}{{TP + TN + FP + FN}} \times 100\% $$

### Datasets

Information on the genetic variants associated with cancers (SNP ID) was retrieved from the GWAS-Catalog database (https:// www.ebi.ac.uk/gwas). Residue change, risk allele frequency, phenotype, and protein accession number were retrieved from The NHGRI GWAS Catalog^20^. The dataset was built after 179,365 genetic variants were filtered based on the keywords' cancer', 'carcinoma', 'glioma', 'leukemia', 'lymphoma', 'melanoma', and 'sarcoma' (Table S2).

### Retrieval of SNPs from the whole-genome sequences

The SNPs that are associated with cancer risks were identified using VCFtools^102^ based on the dataset (Table S2). The variants were then annotated to identify the associated genes, allele frequency (AF), location of the SNPs in the genome sequences, the position of amino acid change in protein sequences, and codon changes using Variant Effect Predictor^103^. hg19/GRCh37 was used as the reference genome for the analyses.

### Identification of the damaging nsSNPs

The functional effects of identified nsSNPS were predicted by using five different bioinformatics tools. These algorithmic programs included Sorting Intolerant From Tolerant (SIFT) [http://sift.jcvi.org/www/SIFT_seq_submit2.html]^25^, Polymorphism Phenotyping v2 (PolyPhen-2) [http://genetics.bwh.harvard.edu/pph2/]^27^, Consensus Deleterious (Condel) [http://bbglab.irbbarcelona.org/fannsdb/query/condel]^50^, Protein Variation Effect Analyzer (PROVEAN) [http://provean.jcvi.org/index.php]^104^, and Protein Analysis Through Evolutionary Relationships (Panther v14.1) [http://www.pantherdb.org/tools/csnpScore.do]^52^. SIFT predicts the effects of an amino acid substitution on protein functions. The sequence homology and the physiochemical characteristics were computed using a normalized probability score (SIFT score) for each substitution^25^. PolyPhen-2 predicts the potential effect of an amino acid substitution on both protein structure and function using a combination of multiple homolog sequence alignment-based methods and protein 3D structure. The prediction is provided as benign, possibly damaging, and probably damaging according to the scores differences of the position-specific independent count (PSIC) between 2 variants (native amino acid and mutant amino acid)^27^. Condel predicts the effect of coding variants on protein function based on the ensemble score of multiple prediction tools (SIFT, PolyPhen-2, FATHMM, and Mutation Assessor)^50^. PROVEAN predicts the functional effects of protein sequence variations, including single or multiple amino acid substitutions and in-frame insertions and deletions^104^. PANTHER estimates the likelihood of a particular nsSNP to cause a functional effect on the protein using position-specific evolutionary preservation^52^. The description of the tools used is presented in Table 1.

**Table 1:** Description of the functional prediction tools.

Program (website)	Algorithm	Input parameters	Evolutionary analysis	Structural attributes	Computing tools	Effect	Score	Prediction	References
SIFT (http://sift.jcvi.org)	Evolutionary conservation	dbSNP rs ID	Multiple Sequences Alignment	/	Matrix Dirichlet	Effect of amino acid substitution on structure/function of protein	0.00—1	< 0.05 = “Damaging” > 0.05 = “Tolerated”	^25^
Polyphen-2 (http://genetics.bwh.harvard.edu/pph2/)	Protein structure/function and evolutionary conservation	dbSNP rs ID	PSIC profiles	Homolog mapping/predictions	Naive Bayesian classifier	Effect of amino acid substitution on structure/function of protein	0.00—1	0.0—0.15 = “Benign” 0.15—1.0 = “Possibly damaging” 0.85—1.0 = “Probably damaging”	^2^
Condel (http://bg.upf.edu/fannsdb/)	Protein structure/function and evolutionary conservation	Genomic coordinate (s), variant(s)	SIFT, PolyPhen-2, MutationAssessor,FATHMM	Homolog mapping/predictions (PolyPhen-2)	Weighted average of the normalized scores from multiple methods	Effect of amino acid substitution on structure/function of protein	0.00—1	0.0 = “Neutral” 1.0 = “Deleterious”	^30^
PROVEAN (http://provean.jcvi.org/index.php)	Evolutionary conservation/ alignment and measurement of similarity between variant sequence and protein sequence homolog	Genomic coordinate (s), variant(s)	BLASTP	/	Blocks Substitution Matrix (BLOSUM62)	Functional effect on protein	(− 40–12.5)	≥—2.5 = “Deleterious” ≤—2.5 = “Neutral”	^104^
PANTHER (http://www.pantherdb.org/tools/csnpScoreForm.jsp)	Evolutionary conservation/ alignment and measurement of similarity between variant sequence and protein sequence homolog	Protein sequences, substitution(s)	Multiple Sequence alignment (PANTHER library)	/	Alignment scores Hidden Markov Models (HMM)	Functional effect on protein	0.00—4200	> 450 = "Probably damaging" 450—200 = "Possibly damaging < 200 = "Probably benign"	^52^
Consurf (https://consurf.tau.ac.il/)	Evolutionary conservation/ alignment and measurement of similarity between variant sequence and protein sequence homolog	Protein sequences (FASTA format), substitution(s)	PSI-BLAST, Multiple sequence alignment (MAFFT (default), PRANK, T-COFFEE, MUSCLE or CLUSTALW)	/	Neighbor-joining Empirical Bayesian or Machine learing, Heuristic algorithm	Evolutionary conservation	1–9	1 = “Most variable positions” (turquoise) 5 = “intermediate conserved positions (white), 9 = “Most conserved positions” (maroon)	^47^
I-Mutant (http://gpcr2.biocomp.unibo.it/cgi/predictors/I-Mutant3.0/I-Mutant3.0.cgi)	Protein stability changes upon single-site mutations from the protein sequence or protein structure	Protein sequences (FASTA format), substitution(s)	Multiple Sequence alignment	Relative Solvent Accessible Area (DSSP Program, DDGMut dataset)	Support Vector Machine (SVM)	Protein Stability changes	DDG > = 0, DDG < 0]	ΔΔG ≤ − 0.5 kcal/mol = ”Destabilizing mutations” ΔΔG ≥ 0.5 kcal/mol = ”Stabilizing mutations” − 0.5 kcal/mol ≤ ΔΔG ≤ 0.5 kcal/mol = “Neutral mutations”	^54^
TM-align (https://zhanglab.ccmb.med.umich.edu/TM-align/)	Alignment and measurement of similarity between two protein structures of known/unknown equivalence	Protein Structure (PDB format)	/	Superposition of two structures (TM-Score)	Heuristic dynamic programming iterations	Protein structure changes	0–1	≤ − 0.5 = “Randomly chosen unrelated proteins” > 0.5 = “same fold in SCOP/CATH”	^69^
ModPred (http://www.modpred.org/)	Post-trasnlational Modification	Protein sequences (FASTA format)	PSI-BLAST	Homolog mapping/ predictions	Position-specific scoring matrices (PSSM)	Identification of PostTtranslational Modification Sites	0–1	> 0.5 = “High confidence” 0.5 = “ Medium” < 0.5 = “Low confidence”	^105^
MutPred2 (http://mutpred.mutdb.org/)	Evolutionary conservation/ alignment and measurement between variant sequence and protein sequence homolog, molecular alterations	Protein sequences (FASTA format), substitution(s)	PSI-BLAST	Homolog mapping/ predictions	Neural network ensemble, Machine learning (ML)	Effect of amino acid substitution and their molecular mechanisms	0–1	≥ 0.5 = “Pathogenic” ≤ 0.5 = “Benign”	^106^

The nsSNPs were considered high-risk if they were predicted to be damaging or deleterious by at least four bioinformatics tools. They were then subjected to further analysis.

### Analysis on conservation of protein evolutionary

ConSurf (consurf.tau.ac.il/) is a bioinformatics tool that was utilized to predict the evolutionary conservation of amino acid in CACFD1, RREB1, LRRC34, ETFA, CPVL, INCENP, FARP2, and TYR protein. It is a web server that builds phylogenetic relationships between homologous sequences to estimate the evolutionary conservation of amino acid positions in a protein or DNA molecule. The conservation analysis on the target proteins was performed to show the significance of each residue position for the protein structure or function. The rate of evolution was determined based on the evolutionary relationship between the protein or DNA, its homologs, and the similarity between amino (nucleic) acids as expressed in the substitutions matrix. Furthermore, Consurf offers an accurate estimation of the evolutionary rate using either an empirical Bayesian approach or a maximum probability (ML) method^47^. Protein sequence in FASTA format was used as the input. UniProtKB accession numbers for the sequences are: CACFD1, Q9UGQ2; RREB1, Q92766; LRRC34, Q8IZ02; ETFA, P13804; CPVL, Q9H3G5; INCENP, Q9NQS7; FARP2, O94887; and TYR, P14679. Consurf created an output consists of the protein sequence and multiple sequence alignment colored by conservation scores. The conservation score ranged from 1 to 9, where 1 to 4 is considered as variable, 5 to 6 as intermediate, and 7 to 9 as conserved amino acid position. We selected those residues with a high score for the high-risk nsSNP for further analysis.

### Analysis of protein stability

I-Mutant Suite is a web server (http://gpcr2.biocomp.unibo.it/cgi/predictors/I-Mutant3.0/I-Mutant3.0.cgi)54 that was used to predict the stability of protein changes caused by a single point mutation. This tool is trained on a ProTherm-derived data set which is the most extensive database on experimental thermodynamic data on free energy changes, which measures protein stability due to mutations^107^. We submitted the protein sequences of selected nsSNPs to predict the impact on the protein stability of the damaging nsSNPs. UniProtKB accession numbers for the sequences are: CACFD1, Q9UGQ2; RREB1, Q92766; LRRC34, Q8IZ02; ETFA, P13804; CPVL, Q9H3G5; INCENP, Q9NQS7; FARP2, O94887; and TYR, P14679. The output included the indicator of the prediction (increase/decrease) of protein stability based on the reliability index (RI) and the predicted Gibbs free energy change (ΔΔG or DDG). The DDG value (kcal/mol) is computed from the unfolding Gibbs free energy value of the mutant protein minus the unfolding Gibbs free energy value of the native protein. The RI ranges from 0 to 10, where 10 is the highest reliability^107^. The free energy change values were categorized into three classes: (i) DDG < − 0.5 kcal/mol as destabilizing mutations; (ii) DDG > 0.5 kcal/mol as stabilizing mutations; (iii) − 0.5 <  = DDG <  = 0.5 kcal/mol as neutral mutations^108^.

### Three-dimensional (3D) protein modeling

The 3D structures of native and mutant (due to nsSNPs) proteins were constructed to explore the differences in the structural stability between the native and mutant proteins. The iterative threading assembly refinement (I-TASSER) server is an integrated platform that provides automated protein structure and function prediction based on the sequence-to-structure-to-function framework^109^. It was employed for the prediction of 3D protein models of native and mutant protein structures with high-risk nsSNPs. It has the most advanced algorithm to build high-quality 3D protein model from amino acid sequences. I-TASSER generates a full-length model of proteins by excising continuous fragments from threading alignments and then reassembling them using replica-exchanged Monte Carlo simulations. SPICKER clusters low-temperature replicas (decoys) generated during the simulation, and the top five cluster centroids are selected for generating full atomic models. The accuracy of the predicted model is reflected in the form of the confidence score (C-score). The C-scores range is between 5 and 2. The greater values of the C-score display higher confidence for the predicted model^109^. The best model for each query protein was selected according to C-score values. Default parameters were used for each of the protein structures. The amino acid sequences of the proteins to be modeled were prepared in the FASTA format as input for the server to predict the native and mutant models. The predicted structures were loaded into PyMOL to visualize their molecular structures. PyMol was used to visualize the molecular structures in high-quality 3D images.

The qualities of all predicted protein structures were then validated by ERRAT tools (https://servicesn.mbi.ucla.edu/ERRAT/)^110^, and Ramachandran Plot. (https://zlab.umassmed.edu/bu/rama/)^111^. ERRAT program analyzed the statistics of noncovalent interactions between three types of atoms, which are carbon (C), nitrogen (N), and oxygen (O). Consequently, six types of interactions are possible (CC, CN, CO, NN, NO, and OO). Ramachandran Plot illustrates the statistical distribution of the combinations of the backbone dihedral angles ϕ and ψ. in protein structures. The number of residues in the allowed or disallowed regions of the Ramachandran plot determines the quality of the model. Template modeling aligns (TM-align) was used for comparison between the predicted native and mutant protein models. Its algorithm identifies the best structural alignment between the protein pairs based on the combination of template modeling-score (TM-score), root means square deviation (RMSD), and the superposition of the structures^69^. TM-score scores range from 0 to 1, where 1 represents the ideal match between two protein structures. In contrast, the higher value of RMSD represents a more significant difference between native and mutant structures.

### Identification of functional and structural properties

MutPred v1.2 and HOPE were used to identify the functional and structural properties of the selected nsSNPs. MutPred is a web application tool that effectively classifies amino acid substitution as being associated with a disease or neutral in human (http://mutpred.mutdb.org/). This tool also helps in predicting the deleterious amino acid substitution or molecular cause of disease^112^. It focuses on a wide range of structural and functional properties, including secondary structure, signal peptide and transmembrane topology, catalytic activity, macromolecular binding, PTMs, metal-binding, and allostery^106^. Protein sequences (FASTA format) of the identified genetic variants and their amino acid substitutions were submitted. MutPred v1.2 generated output scores indicating the probability of deleterious or disease-associated amino acid substitution. The top five features with *P* value impact on the functional and structural properties would be recorded. The predicted scores were classified based on three hypotheses; (i) g > 0.5 and p < 0.05 as actionable hypotheses; (ii) g > 0.75 and p < 0.05 as confident hypotheses; (iii) g > 0.75 and p < 0.01 as very confident hypotheses.

HOPE is a web service tool that was used to identify the structural effects of a point mutation on human protein sequence (www.cmbi.ru.nl/hope)^113^. The protein sequences of the selected nsSNPs were submitted as input. HOPE generated results based on the collected and combined information from several web services and databases. Initially, the algorithm included BLAST against PDB and UniProt to obtain details on the tertiary structure to build a homology model. It was followed by the prediction of the protein features using the Distributed Annotation System^114^.

ModPred (http://www.modpred.org/)^105^ is a web server tool that was used for the prediction of post-translational modification (PTM) sites in proteins based on sequence-based features, physicochemical properties, and evolutionary features. A total of 34 logistic regression models were used in ModPred for 23 different PTM sites to simultaneously predict and analyze multiple types of PTM sites to obtain information on the functional and structural impacts of multiple PTM protein regulatory mechanisms. The 34 ensembles of logistic regression models were trained independently for 23 PTMs on a total collection of 126,036 experimentally tested non-redundant protein sites extracted from various public databases such as SwissProt, HPRD, PDB, Phospho.ELM, PhosphoSitePlus & PHOSIDA and literatures^105^. The PTM sites were predicted to have either low, medium, or high confidence scores. Sites with low confidence have scores of at least 0.5. In contrast, PTM sites with medium and high confidence have different predictor scores that were based on sensitivity and specificity estimates for each of the modifications models as given by ModPred.

### Prediction of protein–protein interactions

STRING is a database and web resource dedicated to protein–protein interactions network, including direct (physical) and indirect (functional) interactions^115^. The database contains data from genomic context, experimental repositories, co-expression, and collections of public text^116^. The available information in the database will allow us to identify and further understand the experimental and/or theoretical interaction for TYR, FARP2, and LRRC34 for this study.

### Molecular docking

The effect of the deleterious point mutations over the binding affinity of FARP2, LRRC34, and TYR, were determined by molecular docking using UCSF Chimera 1.15 tools^60^ with Autodock Vina instruments^61^. Protein and the peptide molecule were given as input for the docking experiments. The protein three-dimensional (3D) crystal structure, MYNN (PDB ID:2vpk), SRC (PDB ID:2h8h), and DCT (PDB ID: 4hx1) from RCSB Protein Data Bank (PDB)^117^ were used as receptors for LRRC34, FARP2, and TYR respectively. The peptide sequences from native and mutant FARP2, LRRC34, and TYR protein structures were used as the ligands for the docking procedure. The peptide sequences of at least nine amino acid residues of each of the native and mutant FARP2, LRRC34, and TYR proteins were converted into Simplified Molecular-Input Line-Entry System (SMILES) strings by using the online tool PepSMI (https://www.novoprolabs.com/tools/convert-peptide-to-smiles-string). The peptide sequences used for the analysis were SGIQQLCDAL, FQGTTKINT, and FEQWLRRHR from native LRRC34, FARP2, and TYR protein and SGIQQICDAL, FQGTNKINT and FEQWLQRHR from mutant LRRC34, FARP2 and TYR protein, respectively. The three-dimensional structure for each ligand was then generated by the Build Structure tool within UCSF Chimera 1.15 software using SMILES as an input. Target proteins and ligands were optimized using the Dock Prep tool from UCSF Chimera 1.15 software^118^ with default parameters before docking analysis. These steps include removing solvents, adding hydrogens, and determining the charge. We maximized the grid box size along with the axes X, Y and, Z accordingly to define the binding sites for conducting the docking. The grid box size was set at 40.4399, 37.7452, 39.3645 along the x, y, and z points, respectively, for MYNN (PDB ID:2vpk) , 69.2063, 68.8481, 75.7427 SRC (PDB ID:2h8h) and 73.99757, 63.0875, 65.1247 DCT (PDB ID: 4hx1). The Autodock from UCSF Chimera 1.15 tools^60^ predicted and evaluated ten (10) protein binding sites for each interaction of receptors and ligands. The same binding sites of native and mutant proteins were compared. The PDB format of these input receptors and ligands were converted into a pdbqt format. The docking result and the binding interaction between ligand and receptor proteins were visualized by UCSF Chimera 1.15 tool.

## Results

### Standard dataset

The dataset contains a total of 100 nsSNPs in which 50 nsSNPS were reported as pathogenic, and 50 nsSNPs were reported as benign (Table S1). The parameters investigated were compared and are presented in Table 2. The sensitivity, specificity, and accuracy of the prediction for the clinical significance of the nsSNPs were calculated for four (4) models (Model A, B, C, and D). Model A represents at least one tool that predicted nsSNPs as deleterious or benign, and it showed the highest sensitivity (100%), followed by Model B (92%), Model C (90%), and Model D (84%). For specificity and accuracy, Model D showed the highest percentages (specificity 94%, and accuracy 89%) followed by Model C (specificity 80%, and accuracy 85%), Model B (specificity 64%, and accuracy 78%), and Model A (specificity 50%, and accuracy 75%). Further analyses were conducted using the combination of five functional effect tools which investigate the conservation and stability (Model A3, B3, C3, and D3). These models resulted in lower sensitivity of deleterious and benign nsSNPs compared to Model A, B, C and D. Interestingly, Model D3 showed the highest specificity (96%) compared to other models (Model A, B, C, D, A3, B3, and C3). However, Model A3, and B3 showed higher accuracy (88%) compared to Model D (89%) and Model C (85%).

**Table 2:** Performance of 8 different prediction models (Model A, B, C, D, A3, B3, C3 and D3) using functional effect prediction tools (SIFT, PolyPhen-2, Condel, PROVEAN, and PANTHER) and conservation (Consurf) and protein stability (I-Mutant). The prediction tools' performance was assessed on a standard dataset with all statistical parameters; TP, FN, TN, FP, sensitivity, specificity, and accuracy.

Statistical parameters	Model A	Model B	Model C	Model D	Model A3	Model B3	Model C3	Model D3
TP (N)	50	46	45	42	48	44	39	38
FN (N)	0	4	5	8	2	6	11	12
TN(N)	25	32	40	47	40	44	46	48
FP(N)	25	18	10	3	10	6	4	2
Sensitivity (%)	100	92	90	84	96	88	78	76
Specificity (%)	50	64	80	94	80	88	92	96
Accuracy (%)	75	78	85	89	88	88	85	86
Annotation	Model A is the combination of five different tools which at least one tool predicted nsSNPs as deleterious/ neutral	Model B is the combination of five different tools which at least two tools predicted nsSNPs as deleterious/ neutral	Model C is the combination of five different tools which at least three tools predicted nsSNPs as deleterious/ neutral	Model D is the combination of five different tools which at least four tools predicted nsSNPs as deleterious/ neutral	Model A3 is the combination of Model A with the prediction of conservation and protein stability	Model B3 is the combination of Model B with the prediction of conservation and protein stability	Model C3 is the combination of Model C with the prediction of conservation and protein stability	Model D3 is the combination of Model D with the prediction of conservation and protein stability

TP = True Positive; FN = False Negative, TN = True Negative and, FP = False Positive.

### SNPs dataset

The database included a total of 3,608 SNPs (excluded redundant nsSNPs entries), 80 are nsSNPs, 21 are sSNPs, 73 in the 3'UTR, 23 in the 5'UTR, 1,922 in the intronic region, 1,078 in the intergenic region, and the remaining are variants in the coding sequence regions, transcription factor binding site, stop- gained region, splice region, regulatory region, splice-acceptor, noncoding transcript and in-frame insertion. The details are provided in the Table S2. For further investigation, only nsSNPs were selected.

### Cancer-related nsSNPs for whole-genome sequences of Orang Asli and Malays

All of the identified SNPs were searched against the SNPs dataset retrieved from GWAS. Out of 80 nsSNPs associated with cancers from the dataset, a total of 52 nsSNPs were found among the Orang Asli and Malays (43 in Orang Asli and 43 in Malays), as presented in Table 3. Thus, we selected all the 52 identified nsSNPs associated with cancer risks among the Orang Asli and Malays for further investigation.

**Table 3:** List of 52 nsSNPs identified among the Orang Asli and the Malays and functional effect predicted by five in silico programs.

SNP ID	Cancer risk	Location	Gene Symbol	Amino acid change	SIFT	PolyPhen-2	ConDel	PROVEAN	PANTHER
rs12621643	Acute lymphoblastic leukemia (childhood)	2:223,917,983	KCNE4	D145E	Tol	benign	Neu	Neu	-
rs13014235	Basal cell carcinoma	2:202,215,492	ALS2CR12	V43L	Tol	benign	Neu	Neu	Prob_ben
rs1050529	Basal cell carcinoma	6:31,324,615	HLA-B	A65T	Del_low_con	benign	Neu	Neu	Prob_ben
rs1126809**	**Basal cell carcinoma or squamous cell carcinoma**	**11:89,017,961**	**TYR**	**R402Q**	**Del**	**Prob_dam**	**Del**	**Neu**	**Prob_dam**
rs11543198*	Bladder cancer	15:74,912,328	CLK3	R78H	Tol_low_con	-	-	Neu	-
rs35273427	Breast cancer	1:120,436,751	ADAM30	T737A	Tol	benign	Neu	Neu	Prob_ben
rs6964587	Breast cancer	7:91,630,620	AKAP9	M463I	Del	benign	Neu	Neu	-
rs1053338	Breast cancer	3:63,967,900	ATXN7	K264R	Tol	benign	Neu	Neu	Prob_dam
**rs3124765**	**Breast cancer**	**9:136,328,657**	**CACFD1**	**I58M**	**Del**	**Prob_dam**	**Del**	**Neu**	-
rs11552449	Breast cancer	1:114,448,389	DCLRE1B	H61Y	Del	benign	-	Neu	Prob_ben
rs3815308	Breast cancer	19:2,226,676	DOT1L	G1386S	Tol_low_con	benign	Neu	Neu	Prob_ben
rs11205303	Breast cancer	1:149,906,413	MTMR11	M159V	Tol	benign	-	Neu	-
**rs9379084**	**Breast cancer**	**6:7,231,843**	**RREB1**	**D1171N**	**Del**	**Prob_dam**	**Del**	**Del**	**Prob_dam**
rs8050871	Breast cancer	16:71,509,796	ZNF19	Q218H	Del	pos_dam	Del	Neu	Prob_ben
rs757978**	**Chronic lymphocytic** **leukemia**	**2:242,371,101**	**FARP2**	**T260N**	**Del**	**Prob_dam**	**Del**	**Del**	**Prob_dam**
rs11539086**	Colorectal cancer	3:58,552,329	FAM107A	E141Q	Tol	Prob_dam	Del	Neu	Prob_dam
rs4836891	Colorectal cancer	9:125,273,574	OR1J2	R165Q	Tol_low_con	benign	Neu	Neu	Prob_ben
rs7248888	Colorectal cancer	19:46,974,003	PNMAL1	C97Y	Tol	benign	Neu	Neu	Prob_ben
rs16845107	Colorectal cancer	3:113,127,991	WDR52	K284N	Tol	benign	-	Neu	Prob_dam
rs3184504	Colorectal or endometrial cancer	12:111,884,608	SH2B3	W262R	Tol	benign	Neu	Neu	Prob_ben
rs1129506	Endometrial cancer	17:29,646,032	EVI2A	S23R	Del_low_con	benign	-	Neu	Pos_dam
rs2278868	Endometriosis or endometrial cancer (pleiotropy)	17:46,262,171	SKAP1	G161S	Tol	benign	Neu	Neu	Prob_ben
rs1229984	Esophageal cancer	4:100,239,319	ADH1B	H48R	Tol	benign	Neu	Neu	Prob_ben
rs671	Esophageal cancer	12:112,241,766	ALDH2	E504K	Del	pos_dam	Del	Del	-
rs2274223	Esophageal cancer	10:96,066,341	PLCE1	H1927R	Tol	benign	Neu	Neu	Prob_ben
rs3765524	Esophageal cancer and gastric cancer	10:96,058,298	PLCE1	T1777I	Tol	benign	Neu	Neu	Prob_ben
rs20541	Hodgkin's lymphoma	5:131,995,964	IL13	Q144R	Tol	benign	Neu	Neu	-
rs3734542*	Lung cancer in ever smokers	6:26,468,326	BTN2A1	R378Q	Tol	benign	Neu	Neu	Prob_ben
**rs10936600**	**Multiple myeloma**	**3:169,514,585**	**LRRC34**	**L286I**	**Del**	**Prob_dam**	**Del**	**Neu**	**Prob_dam**
rs7193541	Multiple myeloma	16:74,664,743	RFWD3	I564V	Tol	benign	Neu	Neu	Prob_ben
rs34562254	Multiple myeloma	17:16,842,991	TNFRSF13B	P251L	Tol	benign	Neu	Neu	Prob_ben
rs1052501	Multiple myeloma	3:41,925,398	ULK4	A542P	Del	benign	Neu	Neu	Prob_ben
rs2272007	Multiple myeloma (hyperdiploidy)	3:41,996,136	ULK4	K39R	Tol	benign	Neu	Neu	Prob_dam
rs6793295	Multiple myeloma and monoclonal gammopathy	3:169,518,455	LRRC34	S249G	Tol	benign	Neu	Neu	Prob_ben
**rs1801591**	**Non-glioblastoma** **glioma**	**15:76,578,762**	**ETFA**	**T171I**	**Del**	**Prob_dam**	**Del**	**Del**	-
rs117744081*	**Non-melanoma** **skin cancer**	**7:29,132,279**	**CPVL**	**Y168H**	**Del**	**Prob_dam**	**Del**	**Del**	**Prob_ben**
rs11170164**	Non-melanoma skin cancer	12:52,913,668	KRT5	G138E	Del	pos_dam	Del	Del	Pos_dam
rs1229984	Oral cavity and pharyngeal cancer	4:100,239,319	ADH1B	H48R	Tol	benign	Neu	Neu	Prob_ben
rs1494961	Oral cavity and pharyngeal cancer	4:84,374,480	HELQ	V306I	Tol	benign	Neu	Neu	-
rs763780	Pancreatic cancer	6:52,101,739	IL17F	H161R	Tol	benign	Neu	Del	Prob_ben
rs2257205	Pancreatic cancer	17:56,448,297	RNF43	R117H	Tol	pos_dam	Neu	Neu	Pos_dam
rs3795244	Pancreatic cancer	17:30,692,396	ZNF207	A240S	Tol	benign	Neu	Neu	Prob_dam
rs130067*	Prostate cancer	HSCHR6_MHC_ MANN:31,163,464	CCHCR1	D275E	Tol	benign	Neu	-	Prob_dam
rs2066827	Prostate cancer	12:12,871,099	CDKN1B	V109D	Tol	benign	Neu	Neu	Prob_ben
rs2277283*	**Prostate cancer**	**11:61,908,440**	**INCENP**	**M506T**	**Del**	**Prob_dam**	**Del**	**Del**	**Prob_dam**
rs2292884	Prostate cancer	2:238,443,226	MLPH	H347R	Tol	benign	Neu	Neu	Prob_ben
rs11071896	Testicular germ cell tumor	15:66,821,250	ZWILCH	S344G	Tol	benign	Neu	Neu	Prob_ben
rs6793295	Thyroid cancer	3:169,518,455	LRRC34	S249G	Tol	benign	Neu	Neu	Prob_ben

Del = Deleterious, Tol = Tolerated, Pro_dam = Probably damaging, Pos_dam = Possibly damaging, Prob_ben = Probably benign, Neutral = Neu,—= Not predicted. *nsSNPs which are found in Orang Asli only. **nsSNPs which are commonly found in Malays only. The highlighted rows were the selected nsSNPs for further investigation.

### Predicted Deleterious nsSNPs among the Orang Asli and Malays

The SNP effect on protein function remains unexplained for a large number of nsSNPs in humans. Five different *in-silico* nsSNPs prediction algorithms were successfully used to predict the impact of all the nsSNPs on the function, structure, and sequence conservation of the proteins in the Orang Asli and Malays studied in this study. The five tools used were SIFT, PolyPhen-2, CONDEL, PROVEAN, and PANTHER. Different algorithms are used by these in silico methods, which often resulted in outputs with different significant values. SIFT prediction scores range from 0 to 1, values less and equal to 0.05 were considered deleterious; all other values are considered neutral. PolyPhen-2 prediction scores range from 0 (benign) to 1 (probably damaging), values near to 1 are more confidently predicted to be probably damaging. PROVEAN predicted variants as deleterious when the score is below the threshold value of − 2.5 and neutral when it is above this value. Besides, Condel predicted the results as deleterious if the score is more than 0.5 and neutral if the score is less and equal to 0.5. PANTHER predicted the length of time (in millions of years) of a position in protein sequence, threshold more than 450 million years is considered as probably damaging, between 450 million years and 200 of millions of years as possibly damaging and less than 200 million years as probably benign. This tool used position-specific evolutionary preservation (PSEP) to determine the length of time a position has been preserved in its ancestors. It would be more likely to have a deleterious impact if the position is in longer preservation. The nsSNPs with greater confidence are expected to be truly deleterious.

In this study, we shortlisted 52 nsSNPs with at least four significant scores out of five algorithmic tools used: score < 0.05 in SIFT, > 0.9 in PolyPhen-2, <  − 2.5 in PROVEAN, 1.0 in CONDEL, and > 450 million years in PANTHER. Therefore, only the most deleterious nsSNPs would be studied. Based on the scores, 6 out of 43 nsSNPs in the Orang Asli and 6 out of 43 nsSNPs in the Malays were shortlisted. Interestingly, four nsSNPs were found in both populations **(**Table 3). As a result, the analysis identified eight deleterious amino acid substitutions responsible for the high-risk nsSNP associated with cancers (Table 3). The nsSNPs which are classified as high risk are rs3124765, rs9379084, rs10936600, rs1801591, rs117744081, rs2277283, rs757978 and rs1126809. They are located on different genes, which are *CACFD1, RREB1*, *LRRC34*, *ETFA*, *CPVL*, *INCENP, FARP2*, and *TYR*, respectively*.* According to the GWAS database, the eight (8) nsSNPs were associated with the risk of specific cancers, as shown in Table 3. Thus, these eight (8) nsSNPs were further investigated.

### Conservation profile of high-risk nsSNPs

ConSurf was further used to investigate the potential impact of the most deleterious nsSNP. It was used to measure the degree of evolutionary conservation of the protein for each amino acid residue. It identifies amino acid positions known to have functional and structural importance through the combination of evolutionary conservation data and solvent accessibility predictions^47^. In this study, all residues of each protein obtained from Consurf were assigned with conservation levels graded with scores ranging from 1 to 9. However, we concentrated only on residues that mapped to the locations of eight (8) high-risk nsSNPs, which we had identified. The server predicted D1171N, I58M, L286, T171I, Y168H, M506T, R402Q, and T260N as highly conserved (Table 4) and their functional and structural importance. The findings further indicated that these eight (8) high-risk nsSNPs were certainly deleterious to the protein functions and structures.

**Table 4:** Conservation profile of amino acids in proteins with high-risk nsSNPs by ConSurf.

SNP ID	UniprotKb Accession Number	Amino Acid Change	Conservation Score	Prediction
rs9379084	Q92766	D1171N	9	Highly conserved
rs3124765	Q9UGQ2	I58M	8	Highly conserved
rs10936600	Q8IZ02	L286I	9	Highly conserved
rs1801591	P13804	T171I	9	Highly conserved
rs117744081	Q9H3G5	Y168H	8	Highly conserved
rs2277283	Q9NQS7	M506T	9	Highly conserved
rs1126809	P14679	R402Q	8	Highly conserved
rs757978	O94887	T260N	8	Highly conserved

1 ≤ conservation score ≤ 4 = variable, 5 ≤ conservation score ≤ 6 = intermediate, and 7 ≤ conservation score ≤ 9 = highly conserved.

### Predicted stability modification

We predicted the stability modifications due to nsSNPs in CPVL, FARP2, CACFD1, RREB1, LRRC34, ETFA, TYR, and INCENP proteins with the help of I-Mutant. The eight (8) nsSNPs that were found associated with cancers were submitted to the I-Mutant 3.0 server to predict the changes in the stability in terms of their free energy change value (ΔΔG) and reliability index (RI). Based on the ΔΔG values, all of these nsSNPs have decreased the stability of the respective proteins (Table 5). However, we had excluded two of them, rs1801591 (RI = 0) and rs117744081 (RI = 4), from analysis as they had RI below five (< 5). The higher RI value shows higher accuracy in the prediction for stability^48^. Thus, the other six nsSNPs (rs3124765, rs9379084, rs10936600, rs2277283, rs757978, and rs1126809) were further analyzed.

**Table 5:** I-Mutant 3.0 and TM-align predictions for nsSNPs associated with cancers among the Orang Asli and Malays.

nsSNP ID	Amino Acid Change	Gene Symbol	Stability	RI	ΔΔG (kcal/mol)	TM-Score	RMSD (Å)
**rs3124765**	**I58M**	**CACFD1**	**Decrease**	**8**	− **1.19**	**0.346**	**4.84**
**rs9379084**	**D1171N**	**RREB1**	**Decrease**	**7**	− **1.74**	**0.319**	**4.41**
rs10936600	L286I	LRRC34	Decrease	5	− 1.00	0.934	2.06
**rs1801591**	**T171I**	**ETFA**	**Decrease**	**0**	− **0.48**	**0.975**	**1.13**
**rs117744081**	**Y168H**	**CPVL**	**Decrease**	**4**	− **1.50**	**0.909**	**2.66**
**rs2277283**	**M506T**	**INCENP**	**Decrease**	**6**	− **0.88**	**0.262**	**2.56**
rs757978	T260N	FARP2	Decrease	5	− 1.01	0.929	3.21
rs1126809	R402Q	TYR	Decrease	9	− 1.39	0.938	2.56

RI = Reliability Index. RMSD = Root Mean Square Deviation. ΔΔG ≤ − 0.5 kcal/mol = destabilizing mutations, ΔΔG ≥ 0.5 kcal/mol = stabilizing mutations, − 0.5 kcal/mol ≤ ΔΔG ≤ 0.5 kcal/mol = neutral mutations. 0.0 < TM-score < 0.30 = random structural similarity, 0 ± 0.3 and 0.5 < TM-score < 1.00 = in about the same fold 0.5 ± 1. Highlighted rows are the excluded nsSNPs.

### Homology modeling of protein

**Figure 2:**
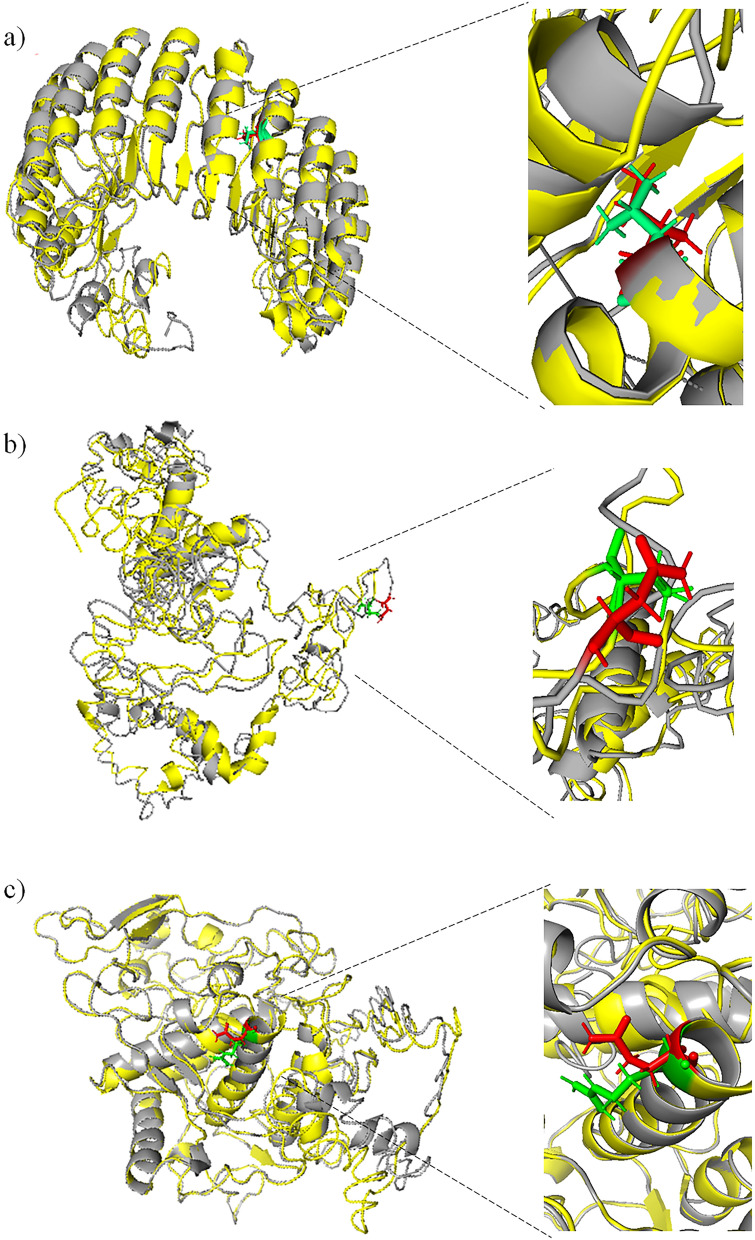
Graphical representations of amino acid changes due to the most deleterious nsSNPs and close-up view for substitution of amino acids (green = native residue; red = mutant residue). (**a**) Superimposed structures of native LRRC34 protein and its mutant with substitution from Leucine to Isoleucine at position 286. (**b**) Superimposed structures of wild type FARP2 protein and its mutant having substitution from Threonine to Asparagine at position 260. (**c**) Superimposed structures of the native TYR protein and its mutant having substitution from Arginine to Glutamine at position 402.

The three-dimensional (3D) structures of 6 native and mutant proteins were predicted by I-TASSER. In generating the mutant models, all six sequences were submitted to the I-TASSER, where each nsSNP was substituted into the native sequence. UniProtKB accession numbers for the native sequences used are LRRC34, Q8IZ02; FARP2, O94887; and TYR, P14679. The available top 10 templates protein models in PDB which are structurally closest to the query protein sequence were used to model the native and mutant proteins of LRRC34, FARP2 and TYR using I-TASSER. Among the six predicted models for each query protein (LRRC34, TYR, FARP2), the best model was selected based on the highest confidence score (C-score), as shown in S3 Table. C-score is the score of confidence for the prediction of pairwise comparison with values ranging from − 5 to 2. A greater level of C-score indicates a model with great confidence and vice-versa. Then, PyMol was used to visualize the structures. Structural analysis of demonstrated that the mutants of LRRC34, FARP2, and TYR had structures with deviated orientation compared to the native LRRC34, FARP2, and TYR, respectively (Fig. 2). Compared to the native structure of LRRC34, FARP2, and TYR proteins, their mutant structures have more helixes as presented in Table 6. The numbers of beta-sheets were also different between the native and mutant proteins. The native protein structure of LRRC34 and TYR have more beta sheets when compared to their mutants. In contrast, the native protein structure of FARP2 has three fewer beta-sheets than its mutant. There are three and two more buried residues in the native LRRC34 (432) and FARP2 (1007) proteins compared to their mutants, respectively. However, buried residues in the native TYR (509) are less than its mutant protein.

**Table 6:** The top 10 templates used for homology modeling, and the alpha helix, beta sheet and exposed/buried residues used by I-TASSER.

	LRRC34		FARP2		TYR	
	L286 (Native)	286I (Mutant)	T260 (Native)	260 N (Mutant)	R402 (Native)	402Q (Mutant)
Templates (PDB id)	1a4yA, 2bnh, 4perA, 4k17A, 6b5bA, 4kxfK, 2p1pB, 3ogmB, 5hywA, 4q62A	4perA, 1a4yA, 1dfjI, 4k17A, 6b5bA, 2p1pB, 3ogmB, 4kxfK, 5hywA, 4q62A	4gzuA, 4h6yA, 3vkhA, 6ez8A, 5xjcA, 3jb9A, 6ar6A, 6bcuA, 5h64A, 5d06A	4gzuA, 4h6yA, 3jb9A, 5xjcA, 2vz8B, 5ganA, 5yz0A, 6bcuA, 5cskA, 5h64A	5m8lA, 4z11A, 5zrdA, 3w6qA, 4ouaB, 6elsA, 3nm8A, 4j3pA, 6hqiA, 4bedB	5m8lA, 3w6qA, 4ouaB, 5zrdA 4z11A, 6elsA, 3nm8A, 4bedB, 4j3pA, 6hqiA
Alpha Helix	15	17	13	14	11	15
Beta sheet	14	13	15	18	11	14
Exposed residue	432	429	1007	1005	509	513
Buried residues	32	35	47	49	20	16

TM-scores and RMSD values of each mutant model were calculated using TM-align. TM-score measures the similarity of topological models for native and mutant proteins, whereas RMSD evaluated the average distance from native α-carbon backbones to mutant models. The mutant model with the highest TM-score value is T171I (0.975), followed by R402Q (0.938), L286I (0.934), T260N (0.929), and Y168H (0.909). The highest TM-score value indicates that the mutant models generated are still in the same folding dimension of the native models but not perfectly the same. Besides, these mutant models were found to be different from the native based on RMSD values shown in Table 5. The nsSNP models of I58M, D1171N, and M506T have very low TM-score values of 0.346, 0.319, and 0.262, respectively, which correspond to randomly chosen unrelated proteins^49^. Hence, we finally selected only three mutants L286I, T260N, and R402Q, for further analysis, based on the results provided by I-Mutant and TM-align (Table 5).

The modeled structures were validated using ERRAT program and Ramachandran Plot Server to check the reliability of predicted protein structures. The ERRAT results showed that the qualities for the native and mutant LRRC34, FARP2, and TYR protein were good with scores of 93.86, 57.17, 75.15, 87.28, 70.27, and 73.51, respectively (Table S3). Ramachandran plots for the native and mutant LRRC34, FARP2, and TYR protein models showed 87.74%, 71.75%, 85.00%, 87.50%, 69.16, and 85.00% of the residues were located in the allowed regions, and only a few amino acids were deviated (Table S3)].

Those three selected mutant protein models were then superimposed on the native protein models to show the location of observed mutations (Fig. 2). The details of the selected native and mutant protein models included the protein templates used to predict the structures and C-score are provided in the Table S3.

### Functional and structural modifications of genetic variants

Three (3) nsSNPs were shortlisted and submitted to the MutPred2 server. MutPred2 predicts the modification of structural and functional protein structures, including the altered order or disordered interface, transmembrane protein, metal binding, DNA binding, loss of allosteric site, and gain of allosteric site. Based on Table 7, the R402Q mutation showed the highest probability score (0.78), followed by T260 mutation (0.73) and L286 mutation (0.55). An amino acid substitution is predicted as pathogenic if a probability score is 0.50 and above.

**Table 7:** Probability scores and top prediction features of deleterious mutations by MutPred2 and ModPred.

Gene Symbol	Mutation	MutPred2		ModPred	
		Top Prediction Features	Score	PTMs	Score
LRRC34	L286I	Altered Ordered interface (*P* value = 0.01) Altered Metal binding (P-value = 0.04)	0.55	Proteolytic cleavage	0.07
FARP2	T260N	Altered DNA binding (*P* value = 2.8e−03) Gain of Allosteric site at F265 (*P* value = 0.03) Altered Disordered interface (P-value = 0.04)	0.70	Proteolytic cleavage	0.49
TYR	R402Q	Altered Disordered interface (*P* value = 0.03) Loss of Allosteric site at R403 (*P* value = 6.3e−03) Altered DNA binding (P-value = 9.1e-03) Altered Transmembrane protein (P-value = 4.6e-03)	0.74	Proteolytic cleavage	0.58

MutPred2: *P* values < 0.05 = confident and *P* values < 0.01 = very confident; MutPred2 score < 0.5 = neutral and MutPred2 score > 0.5 = pathogenic. ModPred scores: < 0.7 = low, ≥ 0.7 = medium, and ≥ 0.9 = high.

**Figure 3:**
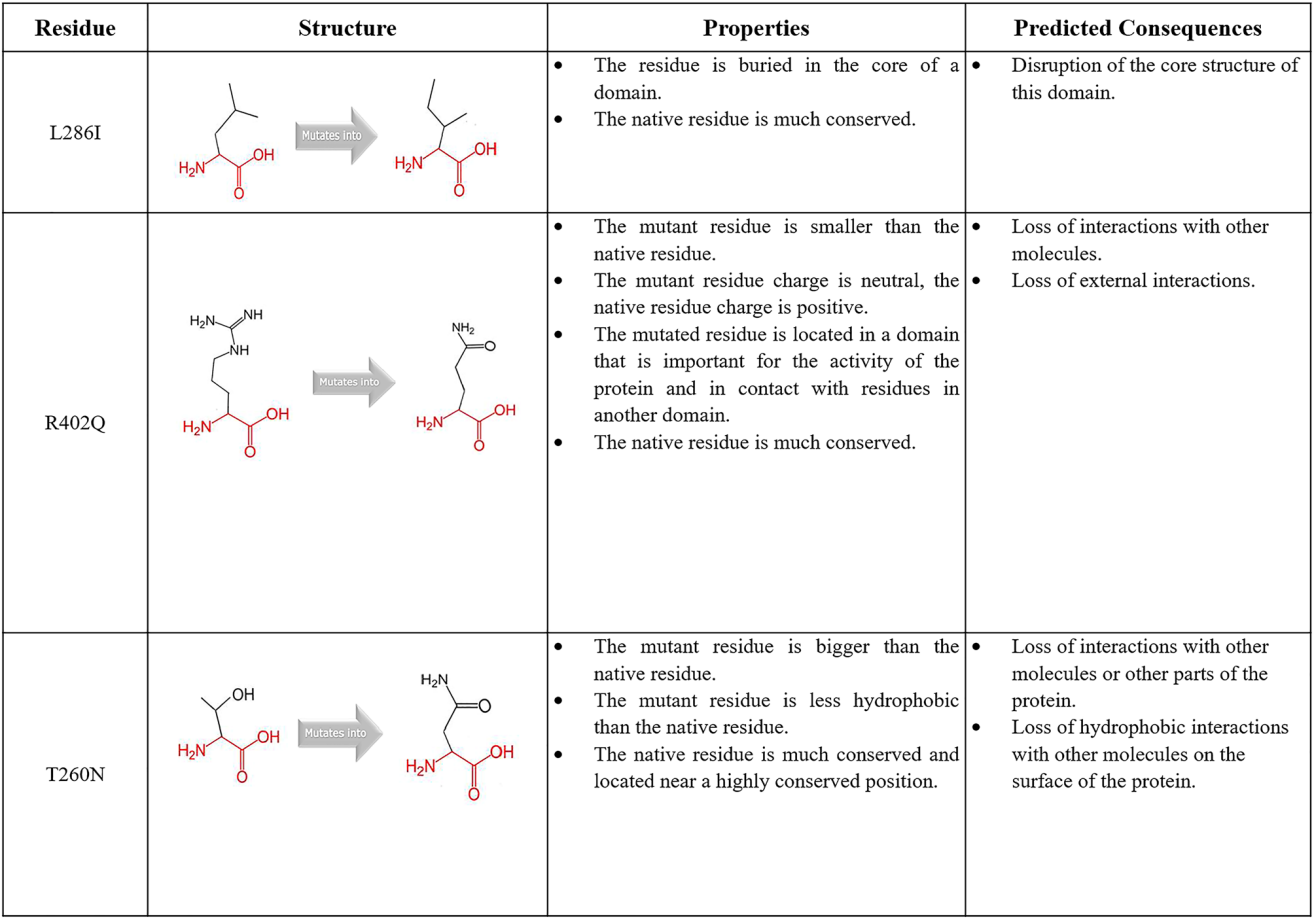
Schematic structures of the original (left) and mutant (right) amino acid for each mutation. The backbone, which is the same for each amino acid, is colored red. The side chain, unique for each amino acid, is colored black. Data obtained from HOPE project.

HOPE was further used to explore the structural effects of these three amino acid substitutions. It was shown that the substitution of L286, T260, and R402 were highly conserved. Based on Fig. 3, the L286I mutation is buried in the core domain, whereas the R402Q mutation was changed to a smaller size amino acid while T260N was changed to a bigger size amino acid than the residue in native protein. Besides, the substitution of amino acid R402Q and T260N had resulted in the change of the net charge of TYR protein and hydrophobicity value of FARP2 protein.

ModPred tools predict possible post-translational modification (PTM) sites to investigate the effects of PTMs on the three substitutions of amino acid L286I, T260N, and R402Q in LRRC34, FARP, and TYR proteins, respectively. Post-translational modifications (PTMs) play a crucial role in regulating many biological processes, such as protein–protein interaction network, protein stability and enzymatic activity, and others. ModPred tool had predicted proteolytic cleavage sites of the substituted amino acids L286I, T260N, and R402Q in LRRC34, FARP, and TYR proteins, respectively (Table 7). Proteolytic cleavage is a PTM that induces activation, inactivation, entirely changed protein structure, excision of new N or C termini with growth factor activity from the parent molecule of an extracellular matrix and regulates a vast range of biological processes. These involve DNA replication, cell proliferation, cell cycle progression, and cells death, and inflammatory processes such as arthritis, cancer, cardiovascular disease, and inflammation. This represents a remarkably significant prediction by ModPred (Table 7).

### Protein–protein interactions analysis

**Figure 4:**
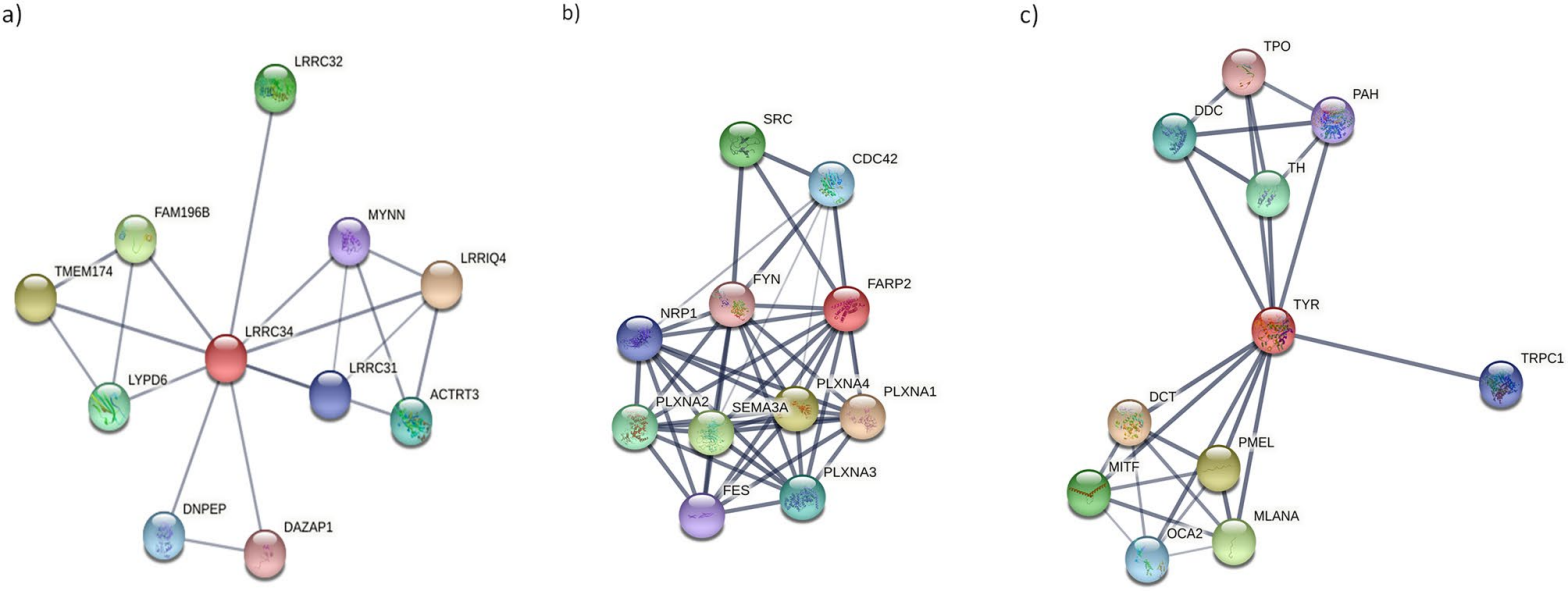
Protein–protein interaction network of proteins; (**a**) LRRC34, (**b**) FARP2, and (**c**) TYR using STRING server. The red node represents the studied proteins.

The STRING server was used to investigate the interaction of HLA-G with various proteins. The interaction analysis revealed that LRCC34 is related to Leucine-rich repeat-containing 32 (LRRC32), Leucine-rich repeat containing 31 (LRRC31), Leucine-rich repeats and IQ motif containing 4 (LRRIQ4), Actin related protein T3 (ACTRT3), Myoneurin (MYNN), Protein FAM196B (FAM196B), Transmembrane protein 174 (TMEM174), Ly6/PLAUR domain-containing protein 6 (LYPD6), Aspartyl aminopeptidase (DNPEP) and DAZ-associated protein 1 (DAZAP1) as shown in Fig. 4.

While FARP2 is related to cell division control protein 42 homolog (CDC42), Proto-oncogene tyrosine-protein kinase Src (SRC), Tyrosine-protein kinase Fyn (FYN), Neuropilin-1 (NRP1), Plexin-A1 (PLXNA1), Plexin-A2 (PLXNA2), Plexin-A3 (PLXNA3), Plexin-A4 (PLXNA4), Semaphorin-3A (SEMA3A), and Tyrosine-protein kinase Fes/Fps (FES) as shown in Fig. 4.

The interaction analysis also revealed that TYR is related to Short transient receptor potential channel 1 (TRPC1), Tyrosine 3-monooxygenase(TH), Phenylalanine hydroxylase (PAH), Aromatic-L-amino-acid decarboxylase (DDC), Thyroid peroxidase (TPO), L-dopachrome tautomerase (DCT), Melanocyte protein PMEL (PMEL), Melanoma antigen recognized by T-cells 1 (MLANA), P protein (OCA), and Microphthalmia-associated transcription factor (MITF) as shown in Fig. 4.

#### Molecular docking analysis

**Figure 5:**
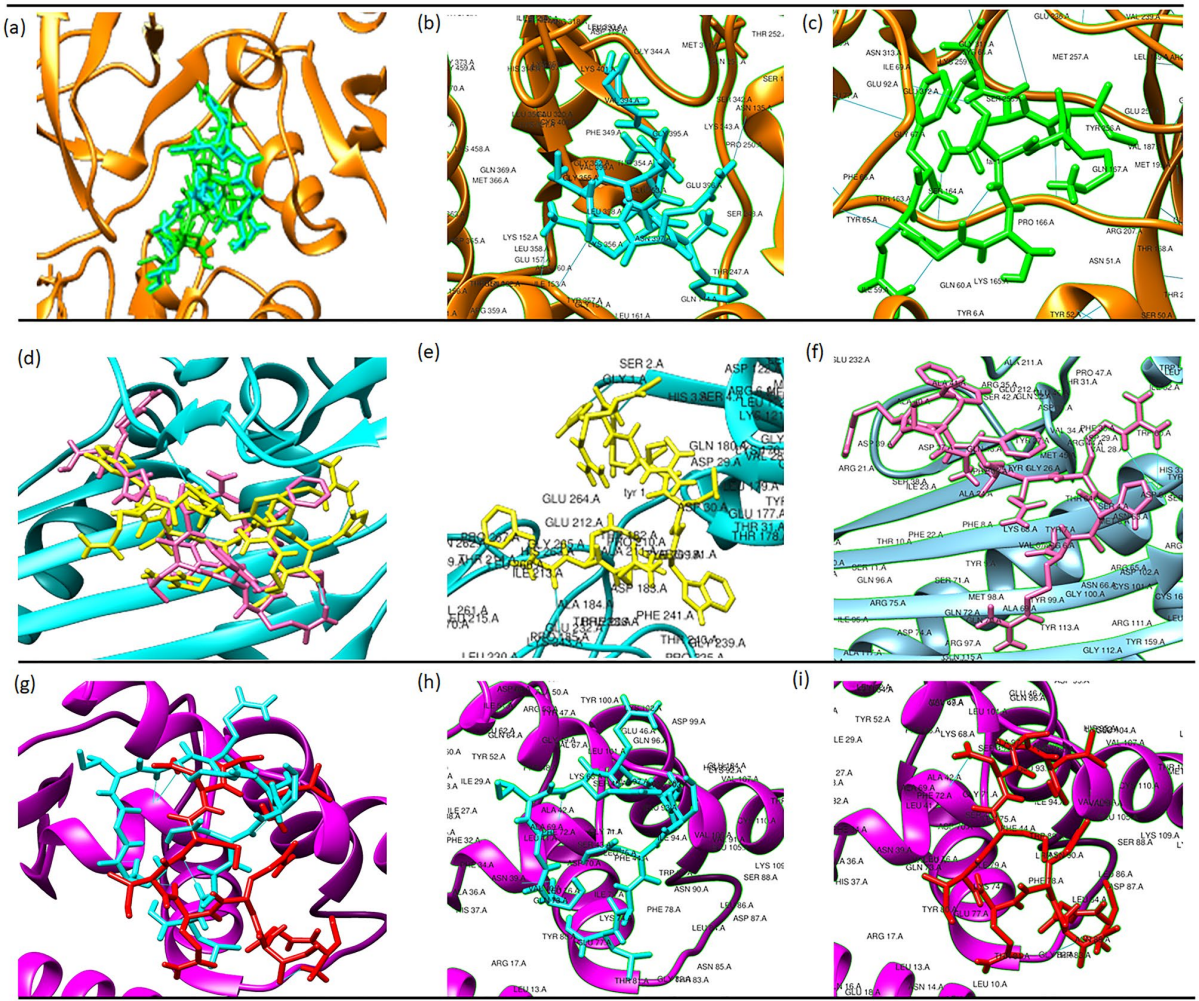
Images of the superimposed native and mutant structural models docked against target proteins with high probabilities values that affect protein functions. (**a**) Superimposed image of SRC (orange) docked against native (blue) and mutant (green) FARP2 protein and interaction of SRC with (**b**) native and (**c**) mutant FARP2 protein structures. (**d**) Superimposed image of DCT (blue) docked against native (yellow) and mutant (pink) TYR protein and interaction of DCT with (**e**) native and (**f**) mutant TYR protein structures. (**g**) Superimposed image of MYNN (purple) docked against native (blue) and mutant (red) LRRC34 protein and interaction of MYNN with (**h**) native and (**i**) mutant LRRC34 protein structures. Hydrogen bonds are presented in a straight blue line.

Autodock Vina, UCSF Chimera 1.15 tools predicted and evaluated a total of 10 protein binding sites along with hydrogen bond interaction and their binding affinities from the docking analysis. The resulting interactions between the native and mutant LRRC34, FARP2, and TYR were compared with those calculated docking results in the same protein binding sites using the exact dimensions of the grid boxes. Thus, a binding site was predicted for each receptor-ligand docking. Molecular docking of SRC, DCT, and MYNN with native and mutant FARP2, TYR, and LRRC34 modeled structures showed differences in the binding affinities (Table 8). The binding affinity of SRC with native FARP2 was − 8.2 kcal/mol, while for mutant was − 7.8 kcal/ mol. The binding affinity of DCT with native TYR was − 8.1 kcal/mol, while for mutant was − 8.0 kcal/mol. The binding affinity of MYNN with native LRRC34 was − 5.4 kcal/mol, while for mutant L286I was 5.2 kcal/mol. In addition, SRC, DCT, and MYNN were bound to the same binding pockets for the native and mutant FARP2, TYR, and LRRC34 proteins, respectively. From the analysis of the binding pose, these three proteins (SRC, DCT, and MYNN) showed significant deviations between the native and mutant protein complexes (Fig. 5**)**. Moreover, interaction analysis of SRC, DCT, and MYNN with the native and mutant FARP2 TYR and LRRC34 proteins showed a reduction in the number of hydrogen bonds with residues in mutant proteins (Table 8). Five residues such as Lys68, Tyr65, Leu5, Ser164, and Gln167 have interactions with SRC in native FARP2 but were absent in mutant proteins. Three residues, Lys152, Ser134, and Lys152, interact with DCT in native TYR but were absent in mutant proteins. Two residues, Asn39 and Ala42, have interactions with MYNN in native LRRC34, but Asn39 was absent in mutant protein.

**Table 8:** Docking results of SRC, DCT and MYNN with native and mutant FARP2, TYR and LRRC34 proteins respectively.

Protein	FARP2				TYR				LRRC34			
	T260	(Native)	N260	(Mutant)	R402	(Native)	Q402	(Mutant)	L286	(Native)	I286	(Mutant)
Binding Affinity (kcal/mol)	− 8.2		− 7.8		− 8.1		− 8.0		− 5.4		− 5.2	
Interacting residue(s) (Hbond)	Residue	Distance	Residue	Distance	Residue	Distance	Residue	Distance	Residue	Distance	Residue	Distance
	Lys68 Tyr65 Leu5 Ser164 Gln167	2.496 Å 1.925 Å 2.467 Å 2.125 Å 1.956 Å	Lys152 Ser134 Lys152	2.366 Å 2.312 Å 1.967 Å	Tyr1 Tyr1 Ser2	1.911 Å 2.384 Å 2.087 Å	Asp29	2.009 Å	Ala43 Gly83	2.025 Å 2.297 Å	Ala42	2.155 Å
Number of hydrogen bond	14		5		8		5		8		3

## Discussion

The exponential increase in the number of nsSNPs detected makes the investigation of the biological significance of each nsSNP by wet laboratory experiments impossible. Alternatively, in silico programs may be used to predict the effects due to mutations and explain the underlying biological mechanisms. nsSNPs in the coding regions can lead to amino acid change and alterations in protein function and account for susceptibility to disease. Identification of deleterious nsSNPs from tolerant nsSNPs is important in analyzing individual susceptibility to disease and understanding disease pathogenesis.

In this study, we have developed a pipeline (Fig. 1) to identify the pathogenic nsSNPs associated with cancers. Although there are various computational tools available to predict the deleterious or damaging effects of nsSNPs on protein structure and function, we had used five different tools (SIFT, PolyPhen-2, Condel, PROVEAN, and PANTHER) to determine the nsSNPs functional effects, while Consurf was used to estimate the evolutionary conservation of the amino/nucleic acid positions in a protein/DNA and protein. I-Mutant 3.0 was used to predict the impact of nsSNPs on the functions or structures of the pathogenic proteins. Among them, SIFT algorithm is the most commonly used tool for SNP characterization to determine deleterious nsSNPs. This method computes a conservation score that provides an insight into the impact of nsSNPs on the functional property of proteins^25^. PolyPhen-2 is considered one of the most reliable tools to predict the functional impact of nsSNPs based on protein sequence, phylogenetic information, and structural information^27^. Condel on the other hand integrates and reflects the combination of scores from different methods (SIFT, PolyPhen2, Mutation Assessor, FATHMM) to classify the nsSNPs. It provides insight into the impact of the mutation on the biological activities of the proteins affected^50^. PROVEAN algorithm is capable of predicting the functional impacts of the amino acid substitution on a protein sequence with commensurable performance and accuracy. It utilizes alignment-based scores to measure the change in sequence variation correlated with the biological function of a protein^51^. Additionally, SIFT, PolyPhen-2, Condel, and PROVEAN, are easy and quick to employ, which allows direct batch queries. Other tools include PANTHER, a powerful and unique method with a curated database of protein families, trees, subfamilies and functions, and evolutionary relationships. It uses phylogenetic trees, multiple sequence alignments, and statistical technique to evaluate the deleterious effects of nsSNPs, making it a viable platform for SNP characterization^52,53^. Consurf is another widely used tool that can pinpoint critically important sites (nsSNPs) within the functional regions. It is a statistically robust approach that estimates the evolutionary rates due to amino acids substitutions and maps them onto the homologous sequence and/or structures^47^. I Mutant 3.0 tool measures the change in protein-free energy caused by a specific mutation^54^. It helps to detect the changes in protein 3D conformation stability. The tools used in this study cover a wide range of prediction techniques (Table 1), combining the findings from each tool in the pipeline will help to identify the most deleterious nsSNPs more accurately. Specific targeted genotyping assays could be developed to detect these nsSNPs identified to be impactful and further investigated in a local cohort of cancer patients. The prediction can also help scientists to focus their study on understanding the impact of these nsSNPs by prioritizing the most deleterious nsSNPs.

The bioinformatics workflow developed was validated using the breast cancer dataset from ClinVar, which acts as a standard dataset. The standard dataset has been annotated and we believe it is the most appropriate dataset for functional effect prediction. The standard dataset contained a total of 100 nsSNPS that were clinically associated with breast cancer (Table S1). The sensitivity, specificity, and accuracy of four models (Model A, B, C, and D) in predicting the clinical significance were determined. Model D represents at least four tools that predicted nsSNPs as deleterious or benign, and it showed the highest percentages of specificity (94%), and accuracy (89%,) followed by Model C (specificity 80%, and accuracy 85%), Model B (specificity 64%, and accuracy 78%) and Model A (specificity 50%, and accuracy 75%). While Model A has the highest sensitivity (100%) followed by Model B (92%), Model C (90%), and Model D (84%). The highest sensitivity scores mean that fewer potentially deleterious nsSNPs were missed. Thus, we concluded that Model D using at least four out of five tools had the best performance in predicting the most deleterious nsSNPs.

Further analyses using the combination of five functional effect tools with conservation and stability tools showed that Model D3 had the highest specificity (96%), but the lowest sensitivity (76%) in identifying deleterious and benign nsSNPs. Despite not having the highest accuracy, Model D3 was able to classify both pathogenic and benign SNVs accurately (86%). The validated workflow is adequate with good sensitivity, specificity, and accuracy to classify the deleterious and neutral nsSNPs in ClinVar using a combination of SIFT, PolyPhen-2, Condel, PROVEAN, PANTHER, Consurf, and I-Mutant.

The GWAS database was used to identify nsSNPs associated with cancer risks as it is the most extensive SNPs database^20^. We only focused on nsSNPs as they are capable of altering protein function, structure, conformation, and interaction which cause the increased risk of cancer^8–10,56–58^. Out of the 80 nsSNPs associated with cancer risks from the GWAS dataset, a total of 52 nsSNPs were identified among the Orang Asli and Malays (43 in Orang Asli and 43 in Malays). They were subjected for further analysis.

Hence, we conducted the concordance analysis with SIFT, PolyPhen-2, Condel, PROVEAN, PANTHER, Consurf, I-Mutant, ModPred, and MutPred tools to predict the most deleterious nsSNPs among the Orang Asli and Malays (Table 3). From the functional effect prediction analysis, a total of 8 out of 52 nsSNPs which were associated with cancers from both populations were identified as the most deleterious nsSNPs by SIFT, PolyPhen-2, Condel, PROVEAN, and PANTHER (Table 3). The most deleterious nsSNPs were identified based on the criteria that at least four scores out of five algorithmic tools used were significant, which are score < 0.05 in SIFT, > 0.9 in PolyPhen-2, <  − 2.5 in PROVEAN, 1.0 in Condel, and > 450 million years in PANTHER. The identified nsSNPs were rs3124765 (*CACFD1*), rs9379084 *(RREB1)*, rs10936600 *(ETFA)* rs1801591 *(LRRC34)*, rs117744081 *(CPVL)*, rs2277283 *(INCENP)*, rs757978 *, (FARP2)* and rs1126809. *(TYR).* In terms of the useability of these five tools for prediction, different algorithms for evolutionary conservation, protein function or structure, alignment, and measurement of similarity between variant sequences and protein sequence homologs were analyzed. Hasan et al.,^59^ had reported that the combination of the best individual tools, FATHMM, iFish, and Mutation Assessor, in one classifier called Meta (Combined Scores through J48 "CSTJ48") enhances the predictive power of these tools. However, no specific classifier outperforms overall datasets in pathogenic predictability. Additionally, these tools have proven performance in identifying deleterious nsSNPs^60,61^, and these make them useful for our study. Thus, these eight (8) nsSNPs identified were further investigated.

The Consurf server had predicted the eight (8) variations, D1171N, I58M, L286, T171I, Y168H, M506T, R402Q, and T260N, were highly conserved (Table 4), and this emphasizes their functional and structural importance. Evolutionary information is essential to understand the mutations potentially affect human health^26^. The evolution of amino acids influence their properties such as size, shape, hydrophobicity, and charge of amino acids at the molecular level^62^. For example, 53 missense mutations that caused cystic fibrosis were found within highly conserved positions. These regions were significant for conserving the structural and functional integrity of the *CFTR* protein^63^. Besides, functional sites of proteins like DNA interaction sites, protein–protein interaction sites, and enzymatic sites are essential for biological functions^64,65^. This may suggest that the nsSNPs found in these conserved regions have higher deleterious effects than other non-conservative nsSNPs and may significantly affected the biological functions^66^. The findings further indicated that these eight (8) high-risk nsSNPs were indeed deleterious to the protein functions and structures.

I-Mutant predicts the protein stability of mutants based on the free energy change value (ΔΔG) and reliability index (RI). I-Mutant predicted 6 out of 8 variants (rs3124765, rs9379084, rs10936600, rs2277283, rs757978, and rs1126809) to have decreased stability. Protein stability is important for the protein structural and functional behavior^67^. Protein stability affects the conformational structure of the protein, such as protein misfolding, aggregation, and degradation, and thus determines its function^67,68^. From the results, we believe that the six variants might had affected the proteins function by affecting their stability.

For structural analysis, the six native and mutant protein structures (CACFD1, RREB1, LRRC34, INCENP, FARP2, and TYR) were successfully generated using I-TASSER as there are no available close homologous templates. I-TASSER generates full-length models by the iterative structural fragment reassembly method, which consistently drives the threading alignment relative to the native state. They were then verified by ERRAT and Ramachandran Plot Server, which proved the stability, reliability, and consistency of the tertiary structures of the proteins. The three-dimensional structures for the native and mutant proteins predicted by I-TASSER clearly revealed the structural changes resulting from amino acids substitutions (Fig. 2). Furthermore, the changes predicted on the sequence-based homology modeling between the native and mutant on the LRRC34, FARP2, and TYR proteins, support the prediction of the pathogenicity of the deleterious substitutions.

TM-align were utilized to calculate the comparison between the predicted native and mutant protein structures based on TM-score and RMSD value. In most cases, common protein structure modeling tools may construct realistic full-length models with an RMSD value less than 6.5 Å if alignment has a TM-score of more than 0.5^69^. Following the criteria of RMSD < 6.5 Å and TM-score > 0.5, three mutants, I58M (CACFD1), D1171N (RREB1), and M506T(INCENP) with TM-scores below 0.5, were excluded. TM-scores below 0.5 correspond to randomly chosen unrelated proteins, meaning that those models were generated from random proteins and had different folding compared to the native protein^49^. Hence, we finally selected only three mutants, L286I (LRRC34), T260N (FARP2), and R402Q (TYR), those with a score higher than 0.5 and which generally assumed the same fold in SCOP/CATH (Table 5). Several studies have shown the importance of using various bioinformatics tools to determine the phenotypic changes and protein function associated with the structure–function relationship of various genes and proteins^70,71^. These studies may provide novel therapeutic markers for a variety of diseases.

The three shortlisted nsSNPs were submitted to MutPred2, HOPE, and ModPred tools to predict the modification of structural and functional protein structures. MutPred2 predicts the modification of structural and functional protein structures, including the altered ordered or disordered interface, transmembrane protein, metal binding, DNA binding, loss of allosteric site, and gain of allosteric site. HOPE was used to further explore the structural effects of these three amino acid substitutions. It was shown that the substitution of L286, T260, and R402 were highly conserved, and they are likely to damage the structures. Based on Fig. 3, the substitution of L286, T260, and R402 caused changes to the LRRC34, FARP2, and TYR protein structures. Modification of protein charge, mass, and hydrophobicity are known to affect the networks of protein–protein interactions^72,73^. Thus, those modifications can alter the ability of proteins to interact with other proteins. Based on these predictions, we believed that several nsSNPs might cause the functional and structural alterations of these proteins and be responsible for the increased risks of cancer. ModPred tools predict possible post-translational modification (PTM) sites to investigate the effects of PTMs further. ModPred tool had predicted proteolytic cleavage sites of the substituted amino acids L286I, T260N, and R402Q in LRRC34, FARP, and TYR proteins, respectively (Table 7). Proteolytic cleavage is a PTM that induces activation, inactivation, fully changed protein structure, excision of new N or C termini with growth factor activity from the parent molecule of an extracellular matrix and regulates a vast range of biological processes. These involve DNA replication, cell proliferation, cell cycle progression, and cells death, as well as inflammatory processes such as arthritis, cancer, cardiovascular disease, and inflammation. This represents a remarkably significant prediction by ModPred (Table 7). The function or structural changes in TYR protein (rs1126809) has been associated with basal cell carcinoma or squamous cell carcinoma. The TYR protein is vital for the production of an enzyme called tyrosinase, which catalyzes the conversion of tyrosine to dopachrome in melanin biosynthesis^74^. We believed that the changes at the PTM site caused by rs1126809 variant of tyrosinase might lead to dysregulation of melanin synthesis within the melanosomes. This resulted in the variation in skin pigmentation, which may lead to basal cell carcinoma or squamous cell carcinoma. As for LRRC34 and FARP2 proteins, the scores given by ModPred for this PTM was very low for proteolytic cleavage (Table 7). The LRRC34 is a nucleolar protein that plays a role in the ribosome biogenesis of pluripotent stem cells. Mutations in some of the related proteins or modifications at ribosome biogenesis may result in severe implications for the organism, depending on the degree of the modification and the involvement of the tissue^75^. The changes at the PTM site might alter the structure of LRRC34 protein, which may lead to multiple myeloma. For example, impaired or modified ribosome synthesis due to the mutation of the ribosomal proteins was reported in many cancers such as chronic lymphocytic leukemia, colorectal cancers, and glioma^76^. FARP2 has been reported as a potential regulator of chronic lymphocytic leukemia pathogenesis that influences protein activity encoded by *MYC* gene. *MYC* gene is known as a proto-oncogene and produces a nuclear phosphoprotein that plays a role in the cell cycle progression, apoptosis, and cell transformation. The mutation may disrupt the MYC protein activity. Although the effect of modification at proteolytic cleavage sites on these proteins has still not been published, numerous studies have shown that this alteration can significantly change the protein function by modifying its position, stability, or inter-protein interactions others^77^. Proteolytic cleavage of modified residues in the protein may be necessary for some of the essential functions of the protein. Besides, those nsSNPs can disrupt proteins that could probably increase the damage caused by PTM impairment.

Protein–protein interaction network analysis showed the interactions of LRRC34, FARP2, and TYR with ten different proteins. This analysis is important in predicting the functionality of interacting genes or proteins and understanding the functional relationships and evolutionary conservation of the interactions among the genes. Besides, our literature search demonstrated that LRRC34, FARP2, and TYR interact with other proteins. LRRC34 interacts with two major nucleolar proteins, Nucleophosmin (NPM1) and Nucleolin (NCL), in ribosome biogenesis of pluripotent stem cells^78^. The mutation in LRRC34 might affects ribosome biogenesis and lead to tumorigenesis. FARP2 interacts with PLXN4, SEMA3A, and NRP1 in Sema3A-Nrp1/PlxnA4 signaling pathway that controls dendritic morphogenesis^79^. The mutation in FARP2 might disrupt the formation of axonal and dendritic morphologies for the neurodevelopment that ultimately lead to risks of cancers. TYR interacts with TH, MITF, and PAH in the melanogenesis pathway^80^. Due to the nonsynonymous mutation in TYR, the melanin synthesis might be disrupted, leading to tumorigenesis. Therefore, any changes in these protein function/structure would have an impact on many disease pathways.

The structural analysis was performed by using molecular docking. The study aims to identify the correct poses of ligands in the binding pocket of a protein and to predict the affinity between the ligand and the protein, which may enhance or inhibit its biological function^81^.

The molecular docking analysis of SRC, DCT, and MYNN with native and mutant FARP2, TYR, and LRRC34 modeled structures showed a difference in binding affinity, reduction in the number of hydrogen bonds with residues in mutant proteins (Table 8), and a significant deviation between native and mutant protein complexes (Fig. 5)**,** respectively. SRC proto-oncogene plays an essential role in development, growth, progression, and metastasis of some human cancers, including those of the colon, breast, pancreas, and brain^82–85^. FARP2 were identified as guanine nucleotide exchange factors (GEFs) for RhoGTPases that play regulatory roles in neuronal development, and several studies have revealed the genetic alterations in Ras homologous RhoGEFs in several human cancers^86–88^. Thus, the deviation observed in the bound SRC molecule with mutant FARP2 protein might disrupt the protein interaction, leading to cancers. A previous study had reported that mutations of melanogenic enzyme tyrosinase (TYR) result in hypopigmentation of the hair, skin and eyes^74^. Besides, DCT is one of the related enzymes that catalyzes different post-TYR reactions in melanin biosynthesis. TYR and DCT also have been proposed to interact with and stabilize each other in multi-enzyme complexes^80^. Thus, the deviation observed in the bound DCT molecule can reduce the catalytic efficiency of TYR. LRRC34 is a member of the leucine-rich repeat-containing protein family that has been suggested to be implicated in the maintenance and regulation of pluripotency. MYNN protein is a member of the BTB/POZ and zinc finger containing family involved in transcriptional regulation. It has also been shown to interact with a few other proteins, including LRRC34, which are part of the transcription factors that participate in DNA repair^89^. A study showed that disruption of LRRC34 protein function could result in reduced expression of some pluripotency genes. Its altered expression impacts the pluripotency-regulating genes and interacts with other proteins known to be involved in ribosome biogenesis^78^. This molecular docking analysis further evaluates our hypothesis as to whether T260N, R402Q, and L238I mutants have deleterious effects on FARP2, TYR, and LRRC34 proteins, respectively. The most prominent change was noticed in T260N, R402Q, and L238I, where a significant loss of H-bond interactions within the binding pocket residues can be observed compared to that in the native protein. These H-bonds were disrupted when the amino acid in mutants was replaced with other amino acids, which altered the binding affinity. The change in the number of hydrogen bonds indicates the deleterious effect of amino-acid substitution. Therefore, an increase or decrease of hydrogen bonds of the native form could destabilize the protein and affect protein functions^90–93^. As a result, genetic mutation which alters the protein structure, therefore influences how the protein interacts with its ligands, potentially leading to a disease condition. This method has previously been used to discover functionally significant variants that may play a role in disease mechanisms^70,94,95^. Molecular docking analysis conducted in this study revealed that T260N, R402Q, and L238I mutants could significantly affect the functional activity of FARP2, TYR, and LRRC34 proteins, respectively.

## Conclusion

With the advancement of genomics, predicting and preventing diseases that are preventable will definitely bring a new facet to medical practice. We had illustrated that with the availability of a local genome database, we could predict disease risks in our population using a validated bioinformatics pipeline and the established GWAS and ClinVar database. The pipeline will help strategize experimental research to prioritize studies on the SNPs with predicted functional impact as thousands and millions of SNPs with unknown functions are detected using whole-genome sequencing technologies.

In this study, a bioinformatics pipeline was developed and validated to predict the effects of nsSNPs, rs1126809, rs757978, and rs10936600 on the functional and structural changes on TYR, FARP2, and LRRC34 proteins, respectively. The analysis also provides significant insight into the deleterious effects of these nsSNPs on the protein structures.

These three (3) nsSNPs were predicted to confer high risks of multiple myeloma, chronic lymphocytic leukemia, and basal cell carcinoma or squamous cell carcinoma in the Orang Asli and Malays population. The prediction pipeline developed in this study helps to reduce the number of extensive investigations and wet lab experiments which are required to explain the impacts of these nsSNPs on the structures and functions of these proteins. We intend to analyze further the risks conferred by these SNPs in the cancer patients in the local population.

We believed that a similar approach could be used to develop and validate bioinformatics pipelines in annotating and predicting the functional effects of SNPs related to other diseases. This study also allows us to establish a database of predicted phenotypes based on the new SNPs identified in our population.

## Supplementary Information

Supplementary Information 1. Table S1. The functional, conservation and protein stability prediction results for 100 nsSNPs associated with breast cancer from ClinVar as a gold standard dataset.

Supplementary Information 2. Table S2. The datasets of nsSNPs associated with cancer, including their ID, gene symbol, protein ID, amino acid change and position, and risk allele frequencies.

Supplementary Information 3. Table S3. The details of native and mutant three-dimensional protein structure models.
