# Supplementary material for: Pathogenic nsSNPs that increase the risks of cancers among the Orang Asli and Malays
Source: Sci Rep. 2021 Aug 9;11:16158. doi: 10.1038/s41598-021-95618-y (PMC8352870; doi:10.1038/s41598-021-95618-y)
Supplement: Supplementary file 3 — Supplementary Information 3. Table S3. The details of native and mutant three-dimensional protein structure models. [file 41598_2021_95618_MOESM3_ESM.docx]

**S3 Table The details of native and mutant three-dimensional protein structure models predicted by I-TASSER, ERRAT score and Ramachandran plot.**

| Gene Symbol | Amino Acid |  | | Native Models | | | | | | | | | Validation | | | | | | | | | | | | |  |
| --- | --- | --- | --- | --- | --- | --- | --- | --- | --- | --- | --- | --- | --- | --- | --- | --- | --- | --- | --- | --- | --- | --- | --- | --- | --- | --- |
|  |  | Templates | **Model 1** | | Model2 | Model3 | | Model4 | | | Model5 | | | ERRAT | | | | | | | Ramachandran  Plot | | | | | |
| LRRC34 | L286 | 1a4yA, 2bnh, 4perA, 4k17A, 6b5bA, 4kxfK, 2p1pB, 3ogmB,  5hywA, 4q62A | **C-score:**  **-0.60**  **TM-score: 0.64±0.13**  **RMSD: 8.5±4.5Å** | | C-score: -2.42 | C-score: -2.46 | | C-score: -3.48 | | C-score: -3.41 | | | | 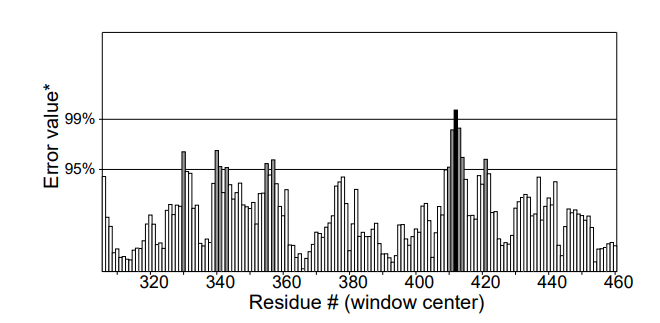  93.86 | | | 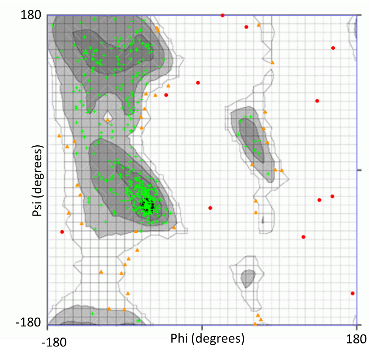  365/416  (87.74%) | | | | | | | |  |  |
| FARP2 | T260 | 4gzuA, 4h6yA, 3vkhA, 6ez8A, 5xjcA, 3jb9A, 6ar6A, 6bcuA, 5h64A, 5d06A, | **C-score:**  **-3.01**  **TM-score: 0.37±0.13**  **RMSD: 16.9±2.8Å** | | C-score: -3.14 | C-score: -3.22 | | C-score: -3.28 | | C-score: -3.17 | | | | 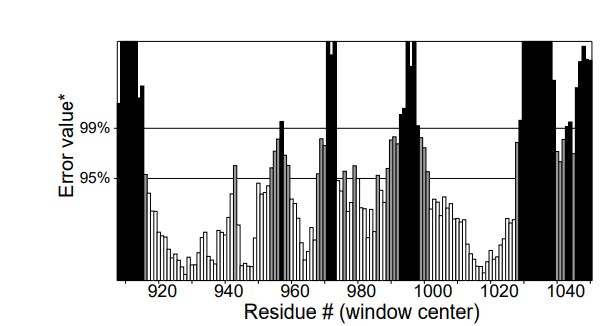  57.17 | | | 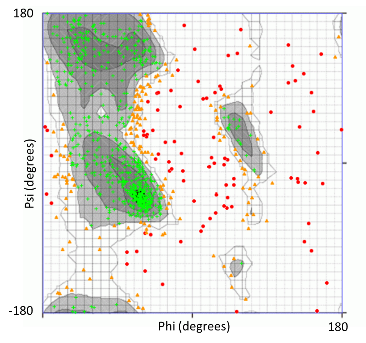  663/924  (71.75%) | | | | | | | |  |  |
| TYR | R402 | 5m8lA, 4z11A, 5zrdA, 3w6qA, 4ouaB, 6elsA, 3nm8A, 4j3pA, 6hqiA, 4bedB | **C-score:**  **-1.06**  **TM-score: 0.68±0.12**  **RMSD: 10.1±4.6Å** | | C-score: -1.14 | C-score: -1.48 | | C-score: -2.39 | C-score: -3.06 | | | 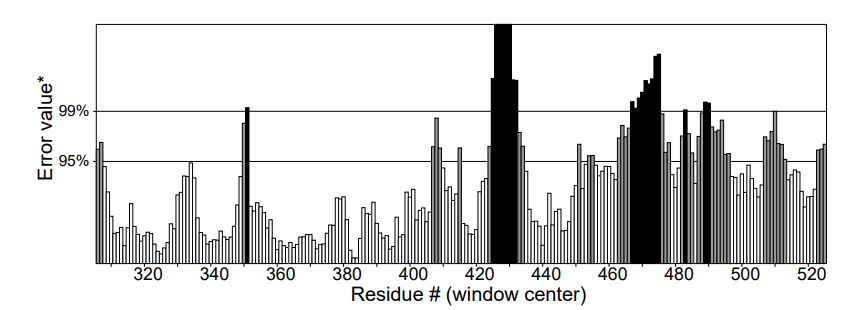  75.15 | | | | | | 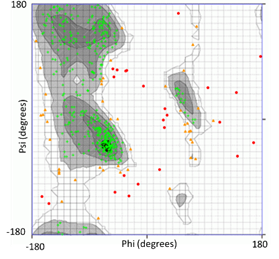  391/460  (85.00%) | | | |  |  |  |  |  |
|  |  |  | Mutant Models | | | | | | | | | | | |  | | | | | | | | |  |  |  |
| LRRC34 | I286 | 4perA, 1a4yA, 1dfjI, 4k17A, 6b5bA, 2p1pB, 3ogmB, 4kxfK, 5hywA, 4q62A | **C-score:**  **-0.24**  **TM-score: 0.68±0.12**  **RMSD: 7.7±4.3Å** | | C-score: -1.52 | | C-score: -2.38 | C-score: -3.18 | | C-score: -1.71 | | | | 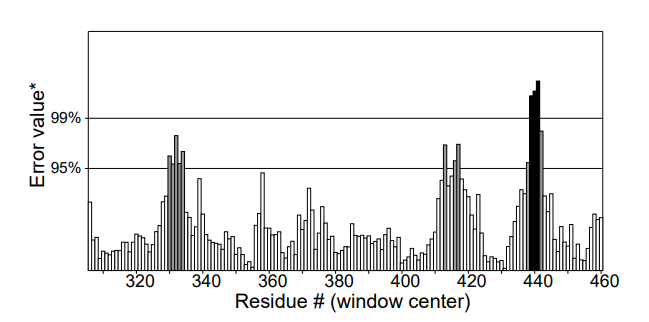  87.28 | | 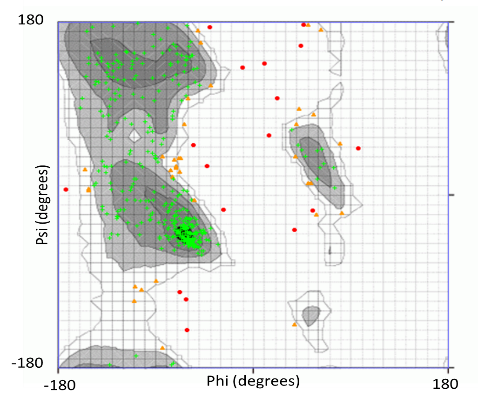  364/416  (87.50%) | | | | | |  |  |  |  |  |
| FARP2 | N260 | 4gzuA, 4h6yA, 3jb9A, 5xjcA, 2vz8B, 5ganA, 5yz0A, 6bcuA, 5cskA, 5h64A | **C-score: -3.02**  **TM-score: 0.37±0.13**  **RMSD: 16.9±2.8Å** | | C-score: -3.15 | C-score: -3.19 | | C-score: -3.25 | | C-score: -3.28 | | | | 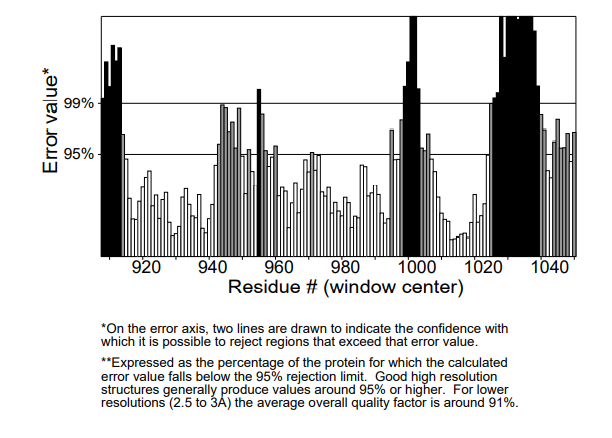  70.27 | | | | | 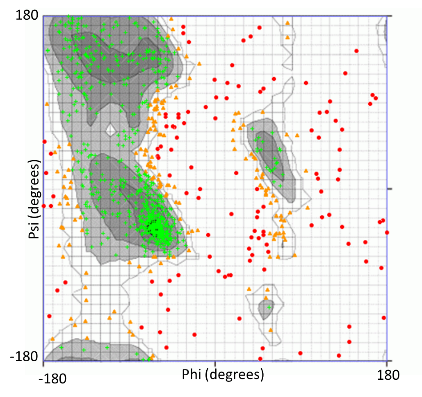  639/924  (69.16%) | | | |  |  |  |  |
| TYR | Q402 | 5m8lA, 3w6qA, 4ouaB, 5zrdA 4z11A, 6elsA, 3nm8A, 4bedB, 4j3pA, 6hqiA | **C-score: -1.29**  **TM-score: 0.54±0.15**  **RMSD: 10.7±4.6Å** | | C-score: -1.39 | C-score: -1.90 | | C-score: -2.22 | | C-score: -2.57 | | | | 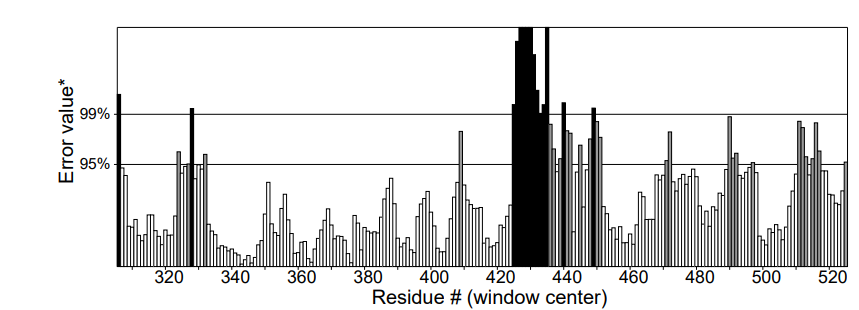  73.51 | | | | | | 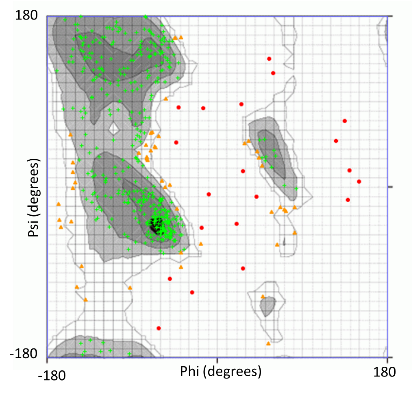  391/460  (85.00%  ) | | |  |  |  |  |

Highlighted column is the best model that were selected based on the highest C-score among the 5 generated models.
